# Mechanisms of action and therapeutic applications of GLP-1 and dual GIP/GLP-1 receptor agonists

**DOI:** 10.3389/fendo.2024.1431292

**Published:** 2024-07-24

**Authors:** Qiyuan Keith Liu

**Affiliations:** MedStar Medical Group, MedStar Montgomery Medical Center, Olney, MD, United States

**Keywords:** GIP, GLP-1, GLP-1RA, tirzepatide, diabetes, obesity, cardiovascular disease

## Abstract

Glucose-dependent insulinotropic polypeptide (GIP) and glucagon-like peptide-1 (GLP-1) are two incretins that bind to their respective receptors and activate the downstream signaling in various tissues and organs. Both GIP and GLP-1 play roles in regulating food intake by stimulating neurons in the brain’s satiety center. They also stimulate insulin secretion in pancreatic β-cells, but their effects on glucagon production in pancreatic α-cells differ, with GIP having a glucagonotropic effect during hypoglycemia and GLP-1 exhibiting glucagonostatic effect during hyperglycemia. Additionally, GIP directly stimulates lipogenesis, while GLP-1 indirectly promotes lipolysis, collectively maintaining healthy adipocytes, reducing ectopic fat distribution, and increasing the production and secretion of adiponectin from adipocytes. Together, these two incretins contribute to metabolic homeostasis, preventing both hyperglycemia and hypoglycemia, mitigating dyslipidemia, and reducing the risk of cardiovascular diseases in individuals with type 2 diabetes and obesity. Several GLP-1 and dual GIP/GLP-1 receptor agonists have been developed to harness these pharmacological effects in the treatment of type 2 diabetes, with some demonstrating robust effectiveness in weight management and prevention of cardiovascular diseases. Elucidating the underlying cellular and molecular mechanisms could potentially usher in the development of new generations of incretin mimetics with enhanced efficacy and fewer adverse effects. The treatment guidelines are evolving based on clinical trial outcomes, shaping the management of metabolic and cardiovascular diseases.

## 1 Introduction

Glucose-dependent insulinotropic polypeptide (GIP) and glucagon-like peptide-1 (GLP-1) are two naturally occurring hormonal peptides produced in gastrointestinal tract, knowns as incretins. Together, they orchestrate a crucial hormonal regulation known as the incretin effect. The concept of incretin effect was first proposed by Creutzfeldt in the 1970s (1, 2), based on the early observations that insulin secretion was two to three times higher after oral glucose intake than that after an isocaloric intravenous glucose administration (3–5). The incretin effect was estimated to account for approximately 50% - 70% of the postprandial insulin responses in healthy individuals and may be substantially reduced to 20% – 30% in individuals with type 2 diabetes mellitus (T2DM) (6, 7), a complex disorder arising from inadequate compensation of insulin secretion by pancreas to counter peripheral insulin resistance. Consequently, researchers have devoted decades to studying incretins, postulating that incretin-based therapies could potentially reverse this diminished incretin effect and restore insulin secretion in patients with T2DM. This review presents a concise history of the discoveries of GIP and GLP-1, explores the physiology and pharmacology of incretins and their synthetic mimetics, and discusses the therapeutic applications of the USA FDA-approved GLP-1 and dual GIP/GLP-1 receptor agonists.

## 2 Physiology of incretins

### 2.1 Discoveries of GIP and GLP-1

**Figure 1:**
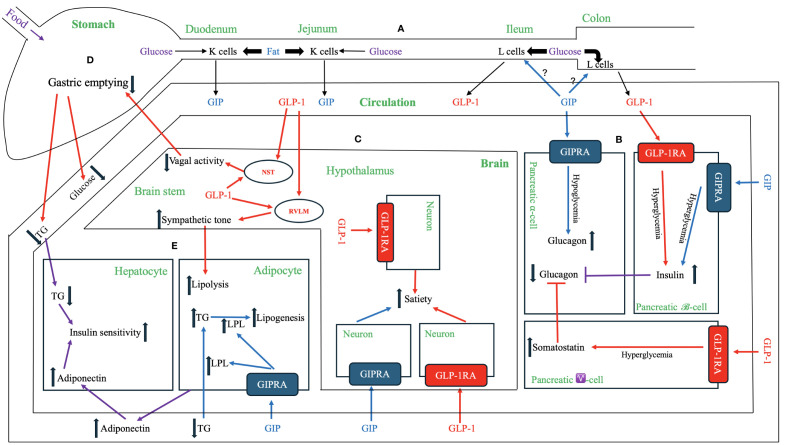
Physiological and Pharmacological Effects of GIP, GLP-1 and Their Receptor Agonists. **(A)** GIP and GLP-1 secretion. Enteroendocrine K cells in the upper gastrointestinal tract (duodenum and jejunum) and L cells in the distal gut (ileum and colon) produce and secret GIP and GLP-1, respectively, into the circulation. GIP peptide and the downstream signaling pathways are depicted in blue, while GLP-1 peptide and the downstream signaling pathways are represented in red. The signaling pathways regulated by both GIP and GLP-1 are shown in violet. **(B)** Effects on pancreatic endocrine functions. Both GIP and GLP-1 have insulinotropic effects on pancreatic β-cells during hyperglycemia. GIP has a glucagonotropic effect in pancreatic α-cells during hypoglycemia but no effect during hyperglycemia. GLP-1 has an indirect glucagonostatic effect during hyperglycemia but no effect during hypoglycemia. **(C)** Effects in the brain. Both GIP and GLP-1 activate their respective receptors in distinct neurons within central nervous system (e.g., hypothalamus) to increase the sense of satiety. Notably, GLP-1, but not GIP, is directly produced in the brain (e.g., nucleus of the solitary tract (NST) and hypothalamus), but both hormones can cross blood-brain barrier from systemic circulation. The central nervous system [e.g., rostral ventrolateral medulla (RVLM)] transmits GLP-1 signaling to increase sympathetic tone, which in turn enhances lipolysis in adipose tissue. The central nervous system (e.g., NST) also relays GLP-1 signaling between afferent and efferent vagal nerves to delay gastric emptying. **(D)** Effects on the stomach. GLP-1delays the gastric emptying, thereby reducing the postprandial lipid and carbohydrate surges. This effect of GLP-1 is at least partially mediated by the neuronal pathway, including afferent vagal nerve, brainstem (e.g., NST), and efferent vagal nerve. GIP does not have a similar mechanism for regulating gastric emptying. **(E)** Effects on adipose tissue and liver. GIP binds GIPR in adipose tissue and activates a signaling cascade that increases lipoprotein lipase (LPL) expression and secretion. LPL enhances triglyceride (TG) clearance from circulation, facilitates the transport of TG to adipocytes for lipogenesis, and attenuates ectopic fat accumulation in visceral organs. GLP-1 indirectly enhances lipolysis via increased sympathetic activity through the central nervous system. Both GIP and GLP-1 have indirect effects on the liver by increasing the secretion of adiponectin from adipose tissue and attenuating hypertriglyceridemia. These mechanisms reduce hepatic fat accumulation, ameliorate insulin resistance, and inhibits hepatic glucogenesis.

**Figure 2:**
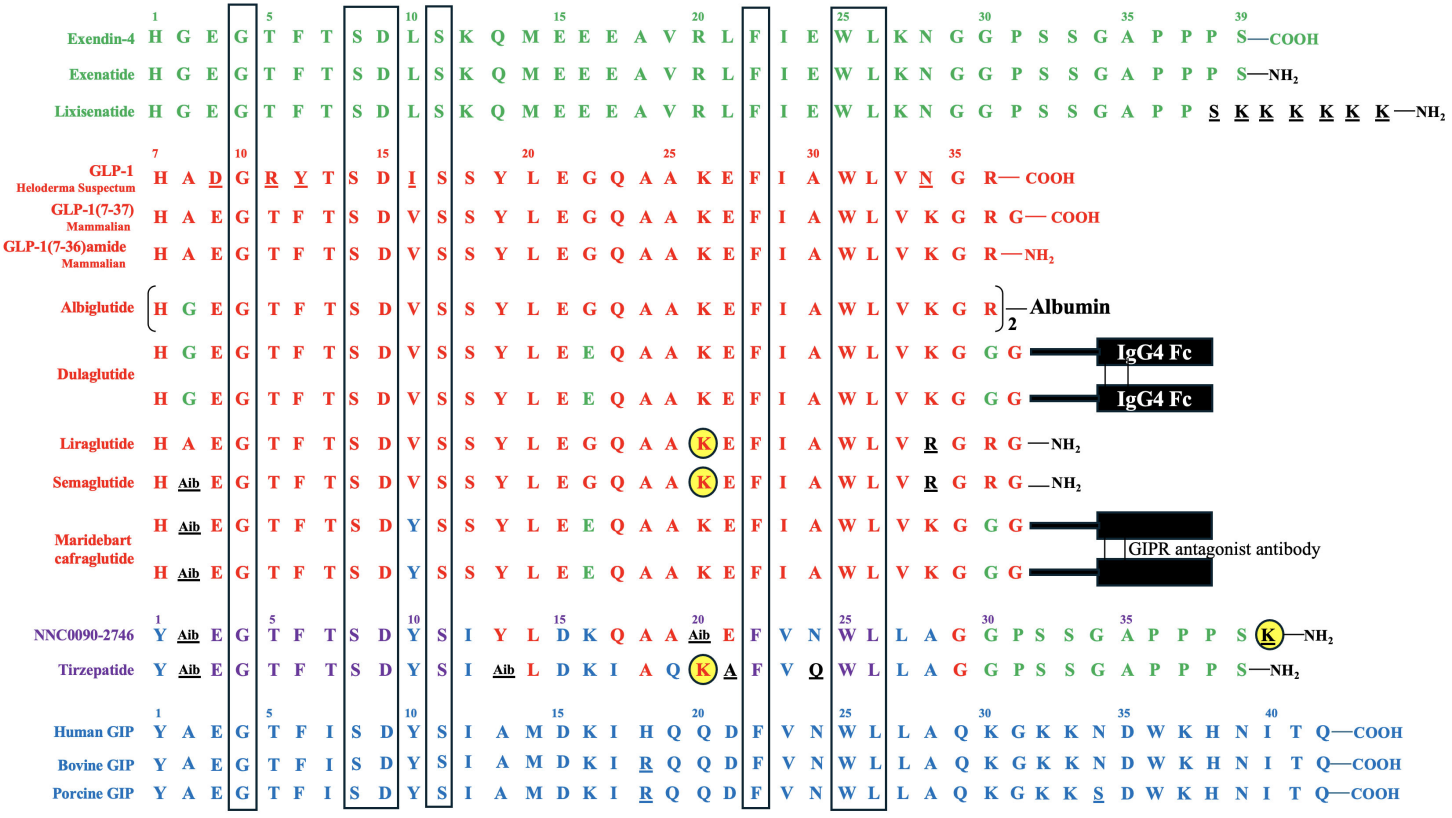
Amino Acid Sequences of Exendin-4, GLP-1, GIP, and Their Derivatives. Exendin-4 and its derivatives are depicted in green, while GLP-1 and its derivatives are represented in red. GLP-1 is conserved across mammalian species. Human GIP and some mammalian (porcine and bovine) GIP variants are shown in blue. The amino acids of the derivatives that differ from their backbone sequences are highlighted in black and underlined. The amino acids of bovine and porcine GIP that are not homologous to human GIP, as well as the amino acids of Heloderma Suspectum GLP-1 that are not homologous to human GLP-1, are also underlined. In NNC0090-2746 and tirzepatide, amino acids shared by both GIP and GLP-1 are displayed in violet, those unique to GIP are shown in blue, those exclusive to GLP-1 are shown in red, and those specific to exendin-4 are displayed in green, while those not identical to GIP, GLP-1 or exendin-4 are depicted in black and underlined. The acylated lysine residues in liraglutide, semaglutide, NNC0090-2746, and tirzepatide are indicated by yellow-filled circles. Seven amino acids conserved in all peptides are highlighted in the boxes.

The first incretin, GIP, was purified from canine intestinal extracts in the late 1960s (8) and initially named gastric inhibitory polypeptide (9) because the peptide inhibited gastrin-stimulated H^+^ secretion (10). In the early 1970s, Dupre et al. (11) discovered that infusion of GIP purified from porcine duodenojejunal mucosa, when combined with glucose, led to enhanced insulin secretion and improved glucose intolerance in humans. As a result, GIP was designated as the first incretin (1, 12). In normal subjects, both fat and carbohydrate stimulate GIP secretion from enteroendocrine K cells, which are dispersed in the upper portion of the gastrointestinal tract (duodenum and jejunum). Interestingly, fat appears to be a stronger stimulator of GIP secretion than carbohydrate (12, 13) [**Figure 1** – Section A]. Most intestinal K cells secrete a biologically active form of GIP consisting of 42 amino acids, GIP (1–42), which is derived from a 153 amino acid preprohormone precursor distinct from preproglucagon (14). GIP is conserved across mammalian species (15); purification and sequencing of porcine and bovine GIP revealed only minor differences (two amino acids in porcine and one in bovine) compared to the human GIP peptide (16–18) [**Figure 2**].

The discovery of the second incretin, GLP-1, originated from observations in 1982 that anglerfish proglucagon mRNA contained coding sequences of glucagon-related peptide, flanked by pairs of basic amino acids characteristic of the sites cleaved during post-translational processing of prohormones (19). In 1983, Bell et al. reported the human and hamster GLP-1 gene and the deduced peptide sequences using cDNA hybridization technology and analysis of the human preproglucagon gene (20, 21). In 1986, Mojsov et al. (22) utilized rabbit anti-serum generated from synthetic peptide to identify GLP-1 peptide in both human pancreatic and intestinal tissues. Meanwhile, Holst et al. (23) employed hydrophobic gel permeation and HPLC technique to isolate GLP-1 from pig ileal mucosa. Subsequently, these two groups independently reported that the synthetic GLP-1 peptide acted as a potent stimulator of insulin secretion in isolated perfused rat pancreas (24) and isolated perfused pig pancreas (23), respectively, thus implicating GLP-1 as the second incretin. This second incretin is produced by enteroendocrine L cells, which are diffusedly distributed in the distal gut mucosa of ileum and colon (25, 26) [**Figure 1** – Section A]. GLP-1 exists in two equipotent circulating peptides: GLP-1 (7–37) and GLP-1 (7–36) amide (27), with GLP-1 (7–36) amide being more abundant in the circulation after a meal (28). The complete conservation of GLP-1 across all mammalian species underscores its critical physiological role (29, 30) [**Figure 2**].

### 2.2 Molecular physiology of GIP and GLP-1

GIP and GLP-1 concentrations appear to be highly variable among individuals, both with and without T2DM. Interestingly, the mean values remain relatively normal in most T2DM groups (31–33), suggesting that the impaired incretin releases are not a typical prerequisite for the development of T2DM. The fasting plasma levels of GIP typically range between 10 – 20 pM with the peak values reaching around 80 – 150 pM after a meal (33–36). On the other hand, fasting plasma levels of bioactive GLP-1 typically fall within the ranges of 5 - 15 pM (33, 37, 38) and could increase 3 – 5 fold postprandially. Under physiological conditions, postprandial GIP levels are approximately 3 – 4 times higher in molar concentration compared to GLP-1, irrespective of diabetic status (28, 39).

Shortly after food intake, these incretins are released into body circulation and bind to their receptors. The physiological effects of GIP and GLP-1 are closely tied to the distribution of their respective receptors in various tissues and organs. Among the tissues and organs crucial in regulating glucose and lipid metabolism, pancreas, brain, and adipocytes express GIP receptors (GIPRs), while pancreas, brain, and gastrointestinal tract are rich in GLP-1 receptors (GLP-1Rs) (40). GIPR was first cloned in rats, identifying a 455-amino acid glycoprotein with a predicted molecular weight of approximately 59 kDa (41). Distinct from GIPR, GLP-1R consists of 463 amino acids and has a molecular weight of 62 kDa (42). Both GIPR and GLP-1R belong to the class B family of 7-transmembrane G protein-coupled receptors (GPCR) within the glucagon receptor superfamily (43). The C-terminal of hormone binds to the extracellular domain, while the N-terminal interacts with the transmembrane domain of the receptor (43). Notably, a study by Finan et al. (44) reported that the half-maximal effective concentrations (EC50) of GIP with GIPR and of GLP-1 with GLP-1R are 20 pM and 28 pM, respectively, without exhibiting cross-reactivity.

The hormonal binding at the extracellular domain is communicated to the intracellular receptor side, leading to G protein engagement and activation (45). The differential insulinotropic potency and other physiological effects of GIP and GLP-1 in both healthy individuals and those with T2DM may be linked to their distinct receptors and the downstream G proteins that transmit signals intracellularly. These pathways exhibit both overlapping function, such as stimulation of adenylate cyclase/cAMP pathway, and unique signaling transduction cascades. Notably, in murine pancreatic β-cells, GLP-1 can activate both G proteins Gαs and Gαq, whereas GIP selectively activates Gαs (45). Despite extensive research into the molecular mechanisms of GLP-1 and GIP actions in pancreatic β-cells, their effects in other cell types remain relatively unexplored.

### 2.3 Interaction of GIP and GLP-1 in the incretin effect

Studies in rodents (46) and canines (47) have delineated the existence of a proximal-distal incretin loop. Within this loop, nutrient stimulation of GIP secretion enhances GLP-1 release [**Figure 1** – Section A]. However, a study by Nauck et al. (48) did not support the existence of this proximal-distal incretin loop in humans as intravenous injection of GIP into humans did not enhance GLP-1 secretion, pointing to the fact that the animal models may differ from humans in essential physiology. Interestingly, individuals with low levels of GIP may also have low levels of GLP-1 and *vice versa* (49). Once secreted, both endogenous GIP and GLP-1 undergo rapid degradation into the biologically inactive metabolites (50, 51). This degradation is catalyzed by the ubiquitous serum enzyme dipeptidyl peptidase 4 (DPP-4), which is produced both locally in the intestine and by circulating white blood cells. Both endogenous GIP (52) and GLP-1 (53) have very short half-lives, measured in minutes, resulting in only a small portion of these active hormones reaching the systemic circulation.

The effects of the incretins with respect to insulin secretion and postprandial glucose regulation have been extensively studied. In healthy individuals, studies indicate that the effects of these two hormones are additive (48, 54). However, the relative contributions of GIP and GLP-1 to the incretin effect and glycemic control under physiological and pharmacological conditions remain a topic of debate. When their respective postprandial concentrations were increased through exogenous infusion, GLP-1 elicited a more robust insulin response, suggesting its superior potency compared to GIP as an incretin (28, 55). Conversely, a study by Gasbjerg et al. (54) found that administering GIPR antagonist to healthy humans reduced postprandial insulin secretion and increased glucose excursions more than administering GLP-1R antagonist (GLP-1RA), indicating that endogenous GIP may play a more significant role in stimulating insulin secretion and reducing postprandial plasma glucose excursions compared to endogenous GLP-1. The relative importance of GIP and GLP-1 may vary depending on meal composition and diabetes stages. Notably, while both carbohydrate and fat ingestions induce considerable increase in GIP secretion, plasma GLP-1 levels primarily surge after glucose-rich meals (56) [**Figure 1** – Section A].

## 3 Pharmacotherapeutic potential of GIP and GLP-1

### 3.1 Therapeutic potential in T2DM

Despite being the first identified incretin, GIP was initially disregarded as a viable therapeutic approach for T2DM. Earlier studies demonstrated that the incretin effect of GIP was severely impaired in patients with uncontrolled T2DM (57); even the pharmacological concentrations of GIP (> 1,000 pM) only marginally stimulate insulin secretion in these patients during hyperglycemic clamp experiments (58). When exposed to a hyperglycemic milieu, the GIPR was found to downregulate in a study of pancreatic islet cells (59) and the patients with T2DM might express a small amount of GIPR or defective GIPR (60, 61). Interestingly, research on clonal pancreatic β-cells revealed that fatty acid load stimulated GIPR expression via the activation of PPAR-α transcriptional factor under normoglycemia (99 mg/dL), but not during hyperglycemia (450 mg/dL) (59). These findings may explain the reduced responsiveness to GIP observed in individuals with T2DM who may have normal or even increased secretion of GIP.

In contrast, while the dose-response relationship between β-cell responsiveness to glucose (expressed as the slope of the linear relation between insulin secretion rate and the glucose concentration) and GLP-1 levels in circulation is partially impaired – a phenomenon referred as “GLP-1 resistance” (62) – a therapeutic infusion of GLP-1 at a pharmacological dose can restore β-cell insulin secretion responsiveness to glucose levels comparable to those observed in nondiabetic individuals (62). A seminal study by Zander et al. in patients with T2DM (38) found that continuous GLP-1 administration via a subcutaneous pump to raise serum GLP-1 levels from a mean of 9.7 pM at baseline to 282 pM over 6 weeks significantly reduced plasma glucose concentrations, improved glycated hemoglobin, and enhanced insulin sensitivity and β-cell function compared to placebo, with no significant limiting side effects reported. These findings provide compelling evidence for the therapeutic promise of GLP-1 in T2DM.

Furthermore, a preclinical study conducted by Finan et al. (44) reported that both GIPR agonist (GIPRA) and dual GIP/GLP-1 receptor agonist similarly improved glucose tolerance in GLP-1R knockout mice, but not in dual incretin receptor knockout mice. In addition, the insulinotropic effect of the dual GIP/GLP-1 receptor agonist was abrogated by GIP receptor antagonist (GIPRA) in GLP-1R null mice (63). These findings underscore the specific GIP action on its receptor in glucose metabolism. GLP-1 action may potentially sensitize GIP signaling, which is often compromised in individuals with uncontrolled T2DM. By facilitating GIP function at its full metabolic capacity alongside GLP-1, this interaction could effectively harness the full potential of incretin effect to enhance insulinotropic physiology and maintain euglycemia.

### 3.2 Therapeutic potential in obesity

Although the incretin effects of GIPR undoubtedly play a crucial role in preventing postprandial glucose excursion, there is still intensive debate on whether the GIPR should be activated or inhibited for the treatment of obesity. Genetic evidence, such as GWAS, has identified the single-nucleotide polymorphism (SNP) in GIPR, showing that lower function of GIPR is associated with lower BMI (64–66), allowing speculation that higher GIPR activity is obesity promoting. In a genetic preclinical study, embryonic GIPR knockout mice fed a high-fat diet were protected from obesity, supporting the role of GIPR antagonism as a method to promote weight loss and prevent weight gain (67). Based on these mouse and human genetic associations, GIPR antagonism has been explored as a therapy to treat obesity (68).

On the other hand, chronically elevating GIP levels in a transgenic mouse model exhibited reduced diet-induced obesity (69). Moreover, a study by Morz et al. (70) demonstrated that treatment with GIPRA led to dose-dependent reductions in body weight in both wild-type and GLP-1R knockout mice, and this effect was negated by co-administration of a GIPR antagonist. Additionally, the weight reduction effect of GIPRA was absent in mice deficient for the GIPR. These findings challenge the prior notion that GIP might be obesogenic and instead suggest that pharmacological activation of GIPR could offer therapeutic benefits for weight reduction. Consequently, these observations have sparked renewed discussions about the therapeutic potential of GIPRAs in the long-term treatment of obesity.

To reconcile the findings that both GIPR antagonists and GIPRAs reduce body weight, it is possible that physiological GIPR activity plays a permissive role in adipose tissue synthesis, while pharmacologically enhanced GIPR activity may reduce body weight through a different mechanism in the brain. The seemingly conflicting effects of GIP on body weight will be discussed further in later sections.

In contrast to the contrasting views on the impact of GIP on the weight, earlier studies unequivocally demonstrated the weight-reducing effects of GLP-1 and GLP-1RAs in both animals and humans. In rats, feeding activity and food intake decreased following intracerebroventricular injection of GLP-1, an effect counteracted by co-administration of a GLP-1 receptor antagonist (71, 72), suggesting central GLP-1 as a potent inhibitor of feeding. Additionally, intraperitoneal administration of GLP-1 at supraphysiological doses elicited an anti-adipogenic effect in rats (72), making GLP-1 a potential target for obesity treatment. A study involving patients with T2DM by Zander et al. (38) revealed that continuous GLP-1 infusion led to significant weight loss over a 6-week period, accompanied by increased sensation of satiety and fullness, along with reduced prospective food intake.

### 3.3 Development of unimolecular agents targeting both GIPR and GLP-1R

The anti-obesity effects of GIPR antagonism antibody, both alone and in combination with GLP-1RAs, have been demonstrated in preclinical models of mice and monkeys (68, 73). Maridebart cafraglutide (previously known as AMG133) is a bispecific molecule engineered by conjugating a fully human monoclonal anti-human GIPR antagonist antibody to two GLP-1 analogue agonist peptides using amino acid linkers. Its half maximal inhibitory concentration (IC50) for GIPR is 42.4 nM, and its EC50 for GLP-1R is 24.4 pM in a human cell-based functional assays (74). In a phase 1 clinical trial, maridebart cafraglutide effectively reduced body weight in participants with obesity (74). The phase 2 study to evaluate the efficacy, safety and tolerability of maridebart cafraglutide is currently ongoing (ClinicalTrials.gov number, NCT05669599).

On the other hand, preclinical studies in rodents have shown that co-administration of a long-acting GIPRA synergistically enhances the glucose-lowering and weight-reducing effects of GLP-1RA (44, 75). Unimolecular peptides with dual GIP/GLP-1 receptor agonism have been developed to improve metabolic efficacy and therapeutic index beyond what incretin mono-agonists can achieve (44, 63). The clinical studies have demonstrated the synergistic effects of GIP and GLP-1 agonism on glucose metabolism and body weight in these unimolecular dual agonists (63, 76), marking a significant achievement in the pharmaceutical development of incretins, which will be further discussed in the next section. Additionally, GLP-1RAs or dual GIP/GLP-1 receptor agonists have been combined with other nutrient-based hormone (e.g., glucagon and amylin) receptor agonists (77–82), a detailed discussion of which is beyond the scope of this review.

## 4 GLP-1 and dual GIP/GLP-1 receptor agonists

The efficacy of native GIP and GLP-1 in treating T2DM has been hampered by their very short half-lives. The current FDA-approved GLP-1RAs are developed as analogues of either exedin-4 or GLP-1. Exenatide and lixisenatide are exendin 4-based agents, while albiglutide, dulaglutide, liraglutide, and semaglutide are GLP-1-based agents. Tirzepatide, the only FDA-approved dual GIP/GLP-1 receptor agonist, is a peptide with potent and imbalanced co-agonism at both GIPR and GLP-1R. The pharmacokinetic differences between short-acting and long-acting analogues have profound implications for the mode of action, efficacy, and tolerability of these compounds. The development of these agents underscores the continuous improvement of the pharmacokinetic and pharmacodynamic profiles of these peptide agents [**Table 1**], expanding their applications beyond T2DM and obesity to encompass cardiovascular disease event reduction [**Table 2**].

**Table 1:** The Peptide Sequences, Formulation Modifications, and Pharmacokinetics of GLP-1 and Dual GIP/GLP-1 Receptor Agonists.

Agent	Backbone sequence	Sequence modification	Covalent or noncovalent formulation modification	Half-life
Exenatide IR	Exendin-4	C-terminal amide	No	2 – 3 hours
Exenatide ER	Exendin-4	C-terminal amide	No	2 weeks
Lixisenatide	Exendin-4	2 amino acids at the C-terminus of exendin-4 are exchanged for 7 different amino acids; C-terminal amide	No	2 -4 hours
Albiglutide	GLP-1 (7–36) — GLP-1 (7–36) —Albumin	Ala8 → Gly	Covalent conjugation with albumin by fusing the two repeats of peptide	6 – 7 days
Dulaglutide	GLP-1(7-37) —linker—IgG4 —linker—GLP-1(7-37)	Ala8→ Gly Gly22 → Glu Arg36 → Gly	Two identical, disulfide-linked chains, each containing an N-terminal GLP-1(7-37) analog sequence and a peptide (Gly_4_Ser)_3_Ala linker, covalently bonded to an IgG4 heavy chain molecule	5 days
Liraglutide	GLP-1(7-37)	Lys34 → Arg C-terminal amide	Acylation at Lys26 with a γ-glutamic acid spacer linked to C16:0	11 - 15 hours
Semaglutide	GLP-1(7-37)	Ala8 → Aib Lys34 → Arg C-terminal amide	Acylation at Lys26 with a γ-glutamic acid-(2,2’-oxydiacetic acid)_2_ spacer linked to C18:0	160 hours
Maridebart cafraglutide (AMG133)	GLP-1(7-37) —linker—GIPR antagonist antibody—linker—GLP-1(7-37)	Ala8 → Aib Val16 → Lys Gly 22 → Glu Arg36→ Gly	2 GLP-1 analogues with the linker [(GGGGS)_3_K(bromoacetyl)(CONH_2_)] conjugated to site-specific engineered cysteines (E384C) to anti-GIPR-Ab	21 days
NNC0090-2746	GLP-1(7-36)/GIP (1-30)	50% homolog with GLP-1; 67% homolog with GIP; C-terminal extension; C-terminal amide	Acylation at Lys40 with C16:0	19 - 25 hours
Tirzepatide	GLP-1(7-36)/GIP (1-30)	50% homolog with GLP-1; 67% homolog with GIP; C-terminal extension; C-terminal amide	Acylation at Lys20 with a γ-glutamic acid-[2-(2-(2-aminoethoxy) ethoxy) acetic acid]_2_ spacer linked to eicosanedioic diacid (C20:0).	120 hours

→, the amino acid at the position number of the peptide on the left side of the arrow is substituted with the amino acid on the right side of the arrow.

**Table 2:** FDA-Approved Dosages and Indications for GLP-1 and Dual GIP/GLP-1 Receptor Agonists.

Agent (trade name)	Maximal dosing regimen approved	FDA-approved indication for T2DM (year of approval)		FDA-approved indication for obesity (year of approval)		FDA-approved indication for cardiovascular risk reduction (year of approval)
		Adult	Adolescent	Adult	Adolescent	
Exenatide IR (Byetta®)	10 μg twice daily injection	Yes^1^ (2005)	No	No	No	No
Exenatide ER (Bydureon®)	2 mg once weekly injection	Yes (2012)	No	No	No	No
Lixisenatide^2^ (Adlyxin®)	20 μg once daily injection	Yes (2016)	No	No	No	No
Albiglutide (Tanzeum®^3^)	50 mg once weekly injection	Yes (2014)	No	No	No	No
Dulaglutide (Trulicity®)	4.5 mg once weekly injection	Yes (2014)	No	No	No	Yes^4^ (2020)
Liraglutide^5^ (Victoza®, Saxenda®)	Victoza®: 1.8 mg once daily injection for T2DM Saxenda®: 3.0 mg once daily injection for obesity	Yes (2010)	Yes (≥ 10 y/o) (2019)	Yes (2014)	Yes (≥12 y/o) (2020)	Yes^6^ (2017)
Semaglutide (Ozempic®, Wegovy®)	Ozempic®: 2 mg once weekly injection for T2DM Wegovy®: 2.4 mg once weekly injection for weight management	Yes (2017)	No	Yes (2021)	Yes (≥12y/o) (2022)	Yes^6^ (2020)
Oral semaglutide (Rybelsus®)	14 mg once daily oral	Yes (2019)	No	No	No	No
Tirzepatide (Mounjaro®, Zepbound®)	Mounjaro®: 15 mg once weekly injection for T2DM Zepbound®: 15 mg once weekly injection for weight management	Yes (2022)	No	Yes (2023)	No	No

^1^As the first GLP-1RA approved for type 2 diabetes.

^2^Monotherapy is no longer available in the USA since Jan 2023, and lixisenatide-glargine (iGlarLixi®) has been approved for type 2 diabetes.

^3^In July 2018, the manufacturer removed albiglutide from the market due to financial reasons.

^4^Approved to reduce the risk of major adverse cardiovascular events (cardiovascular death, nonfatal myocardial infarction, or nonfatal stroke) in adults with type 2 diabetes mellitus and established cardiovascular disease or multiple cardiovascular risk factors.

^5^Insulin degludec plus liraglutide (IDegLira®) has been approved for type 2 diabetes mellitus.

^6^Approved to reduce the risk of major adverse cardiovascular events (cardiovascular death, nonfatal myocardial infarction, or nonfatal stroke) in adults with type 2 diabetes mellitus and established cardiovascular disease.

### 4.1 Exendin 4-based agents

Exendin-4, a 39-amino acid peptide with 53% sequence homology to human GLP-1 [**Figure 2**], was originally isolated from *Heloderma suspectum* lizard venom by using an amino acid sequencing assay targeting peptides with an amino-terminal histidine residue (His^1^) (83). Although 16 of the 30 amino acids in its N-terminus are identical to human GLP-1 (7–36) amide, exendin-4 is not the lizard homolog of GLP-1. In comparison, lizard GLP-1 itself shares an 83% sequence identity to human GLP-1 (84) [**Figure 2**]. The shared biological properties of exendin-4 and human GLP-1 probably stem from their primary and secondary structures, but exendin-4 is naturally resistant to degradation by the DPP-4 enzyme, attributed to the presence of glycine at position 2, resulting in an intravenous half-life of approximately 30 minutes (85). In cellular assays, exendin-4 exhibits similar potency as human GLP-1 in binding to and activating GLP-1R (86). However, in murine models, exendin-4 demonstrates about 5,500-fold greater potency in improving glucose control, evidenced by the percentage fall in plasma glucose at 1 hour (87). The serendipitous discovery of exendin-4 led to the development of exenatide immediate-release (exenatide IR, Byetta®), a synthetic version of exendin-4 with C-terminal amidation to enhance its stability [**Figure 2**, **Table 1**], which became the first FDA-approved GLP-1RA in 2005 for the treatment of T2DM in adults [**Table 2**].

Lixisenatide is a 44 amino acid synthetic peptide derivative of exendin-4 [**Figure 2**, **Table 1**]. Binding studies in CHO-K1 cells expressing GLP-1R showed that lixisenatide is a potent and selective GLP-1RA, with a binding affinity to GLP-1R approximately four times greater than that of GLP-1 (88). Despite its relatively short half-life (2 – 4 hours), lixisenatide is recommended for once-daily dosing in the treatment of T2DM [**Table 2**]. However, as of 2023, lixisenatide is no longer available as a standalone agent in the USA; it is now only available in the combination formulation of insulin glargine plus lixisenatide (iGlarLixi ®).

A long-acting release form of exenatide (exenatide ER, Bydureon®) was approved by the FDA in 2012, becoming the first once-weekly injection of a GLP-1RA for adults with T2DM [**Table 2**]. The formulation of exenatide ER comprises the encapsulation of exenatide IR peptides within 0.06 mm-diameter injectable microspheres made of poly-(D,L lactide-co-glycolide) – a biodegradable medical polymer enabling controlled, gradual drug release over an extended period (89). As the polymer hydrolyzes, it steadily releases the encapsulated exenatide peptides over a maximal duration of 7 weeks. The breakdown products – lactic acid and glycolic acid – are subsequently eliminated from the body as carbon dioxide and water.

### 4.2 GLP-1-based agents

The focus of GLP-1RA development has predominantly been on designing GLP-1 derivatives resistant to DPP-4 enzymatic activity and exhibiting slow renal clearance through chemical modification and pharmaceutical formulation. These approaches are aimed at extending their pharmacological half-lives and attaining supraphysiological stimulation of GLP-1R.

Both albiglutide and dulaglutide are GLP-1 analogues with an alanine-to-glycine substitution at position 8, rendering the peptides resistant to DPP-4 enzymatic activity [**Figure 2** and **Table 1**]. Albiglutide is generated from a genetic fusion of two modified recombinant GLP-1 (7–36) molecules linked in tandem to recombinant human albumin (90), while dulaglutide is created by conjugating two GLP-1 (7–37) analogues to the Fc fragment of a modified monoclonal antibody (IgG4) via peptide ((Gly_4_Ser)_3_Ala) linker (91). The large molecular entities of both albiglutide and dulaglutide delay their renal clearance (92, 93). Albiglutide was less effective in activating the GLP-1R compared to exendin-4 in an *in vitro* study of a BHK cell line expressing the rat GLP-1R (92). In another study, dulaglutide demonstrated full receptor activity *in vitro* and elicited insulinotropic effects in islets similar to GLP-1 (94). Both albiglutide and dulaglutide received FDA approval for the treatment of T2DM in 2014, with dulaglutide having an additional indication since 2020 to reduce the risk of major adverse cardiovascular events (MACE) in adults with T2DM and established cardiovascular disease or multiple cardiovascular risk factors [**Table 2**].

Once daily injectable liraglutide (marketed as Victoza® for T2DM and Saxenda® for obesity) and once weekly injectable semaglutide (marketed as Ozempic® for T2DM and Wegovy® for obesity) are two additional GLP-1 analogues, sharing 97% and 94% amino acid homology to GLP-1, respectively [**Figure 2**, **Table 1**]. Both employ fatty acid acylation of lysine 26 to facilitate serum albumin noncovalent binding to prolong their plasma half-lives (95). Semaglutide features a substitution of alanine residue with a non-coded amino acid, 2-aminoisobutyric acid (Aib) at position 8, shielding against DPP-4-mediated N-terminal proteolysis while preserving GLP-1R affinity (96). The crystal structures of un-acylated liraglutide and semaglutide are almost identical to GLP-1 (7–37). Pharmacokinetic data from a clinical trial involving adolescent participants treated with 3.0 mg liraglutide daily showed a median liraglutide concentration of 29.4 nM at week 8, declining to 17.7 nM at week 56 (97). In a population pharmacokinetic analysis of four clinical trials, the mean semaglutide plasma concentration was estimated at 15.3 nM with 0.5 mg/week of semaglutide and 30.6 nM with 1.0 mg/week of semaglutide in the blood samples collected from week 4 to week 56 of treatment (98). Liraglutide is equipotent to GLP-1 in activating GLP-1R, with an EC50 of 61 pM for liraglutide vs. 55 pM for GLP-1 in one cellular assay (99). In another study, semaglutide exhibited greater potency in activating GLP-1R compared to GLP-1, with an EC50 of 6.2 pM for semaglutide vs 16.2 pM for GLP-1 (95) (The variability in EC50 values among different studies for GLP-1 was not uncommon attributable to varying assay conditions). Both liraglutide and semaglutide have received FDA approval for the treatment of T2DM, obesity, and cardiovascular risk reduction [**Table 2**]. In addition, liraglutide combined with insulin degludec (IDegLira®) is a once-daily, fixed dual combination product approved for T2DM.

Peptide degradation in the gastrointestinal tract poses a significant challenge in the development of oral formulations of incretin analogues. An oral formulation of semaglutide (Rybelsus®) is the first oral GLP-1RA approved for treating T2DM [**Table 2**]. In this oral formula, semaglutide is non-covalently linked to sodium N-[8-(2-hydroxybenzoyl) aminocaprylate] (SNAC), which shields the peptide from enzymatic and acidic degradation in the stomach (100). The absorption is compound-specific and transcellular, facilitated by SNAC (100). Despite its oral bioavailability being less than 1%, this approach is therapeutically feasible because of the strong potency of semaglutide for GLP-1R activation (101). In a 10-week clinical trial, a daily dose of 20 mg oral semaglutide achieved a steady-state plasma concentration of 30 nM, and when daily dose of oral semaglutide was increased to 40 mg, the semaglutide plasma concentration reached about 60 nM (102), which was over a thousand times higher than the EC50 of semaglutide for GLP-1R activation (95). After absorption, the pharmacokinetic properties and effects of semaglutide are similar, irrespective of the route of administration (103). The half-life of oral semaglutide is approximately 1 week, aligning with subcutaneously administered form (102). The highest approved dose of oral semaglutide (14 mg/day) is currently indicated only for treating T2DM as an adjunct to diet and exercise. Higher doses of oral semaglutide (up to 50 mg/day) are under development for the treatment of both T2DM (104) and obesity (105), with effectiveness on par with 2.4 mg/week subcutaneous semaglutide approved for weight management.

### 4.3 Dual GIP/GLP-1 receptor agonists

The unimolecular dual incretin receptor agonist offers a more physiological approach to managing diseases associated with T2DM compared to GLP-1RAs alone. To date, clinical data have been reported for two dual GIP/GLP-1 receptor agonists. The first dual GIP/GLP-1 receptor agonist, NNC0090-2746 (also known as RG7697, RO6811135, or MAR709), is a 40-amino acid peptide acylated with a C16:0 fatty acid to lysine residue at position 40 [**Figure 2**, **Table 1**]. The modified peptide contains 2 Aib substitutions at positions 2 and 20 with a molecular weight of 4.5 kDa. It possesses *in vitro* balanced GIPR (EC50 = 3 pM) and GLP-1R (EC50 = 5 pM) agonism with relative activity seven times that of GIP to GIPR and five times that of GLP-1 to GLP-1R, respectively (44). Pharmacokinetics of NNC0090-2746 showed a C_max_ at 5.4 nM with a single dose of 1.8 mg (106) and C_max_ at 12 nM at the steady state with a daily dose of 2 mg in patients with T2DM (107).

The second dual GIP/GLP-1 receptor agonist, tirzepatide (previously known as LY3298176; marketed as Mounjaro® for T2DM and Zepbound® for obesity), is a 39-amino acid peptide acylated with a C20:0 fatty diacid moiety to lysine residue at position 20 with a molecular weight of 4.8 kDa [**Figure 2**, **Table 1**]. Tirzepatide is engineered as an imbalanced agonist in terms of its strong affinity and potency at the GIPR versus the GLP-1R. Preclinical data indicated that tirzepatide exhibited an affinity for GIPR equivalent to GIP binding, while its affinity for GLP-1R was approximately five times weaker compared to GLP-1 (63). In signaling studies using cell lines expressing GIPR or GLP-1R, tirzepatide demonstrated similar potency to GIP in activating GIPR (EC50 = 22.4 pM for tirzepatide vs. 33.4 pM for GIP) but approximately 13-fold weaker potency than GLP-1 in activating GLP-1R (EC50 = 934 pM for tirzepatide vs.70.5 pM for GLP-1). Pharmacokinetics of tirzepatide demonstrated dose proportionality over the wide dose range, with C_max_ of 180 nM in individuals treated with one dose of 8 mg and a C_max_ of 260 nM with 15 mg/week dose in the clinical study, exceeding the EC50 values for GIPR and GLP-1R activations by approximately 10,000-fold and 250-fold, respectively (63).

The acylation of lysine residues in NNC0090-2746 and tirzepatide enables noncovalent albumin binding and prolongs their renal clearance [**Figure 2**]. However, tirzepatide has significantly longer half-life than NNC0090-2746 in humans (120 hours vs. 19 – 25 hours) [**Table 1**]. NNC0090-2746 and tirzepatide also display unique agonism properties at their target receptors (108). In the cellular studies, NNC0090-2746 and tirzepatide showed comparable efficacy and potency at multiple signaling pathways connected to the GIPR. Tirzepatide, compared to both GLP-1 and NNC0090-2746, shows biased signaling at GLP-1R, favoring cAMP response over β-arrestin recruitment, which leads to low efficacy for GLP-1R internalization (108–110). It remains unclear whether the imbalanced pharmacology or the longer half-life of tirzepatide contributes to its superior efficacy in glucose and weight reduction compared to the more balanced dual agonist, NNC0090-2746.

Both NNC0090-2746 (107) and tirzepatide (63) delivered clinically meaningful improvements in glycemic control and body weight reduction in their phase 1 trials. In the phase 2b trial, however, the reductions in glycated hemoglobin and body weight in participants treated with NNC0090-2746 were similar to those in the group treated with liraglutide (76), and no phase 3 trial of NNC0090-2746 has been reported yet. Conversely, in another phase 2b study involving patients with T2DM, tirzepatide showed significantly better efficacy in glucose control and weight loss compared to both placebo and dulaglutide, with an acceptable safety and tolerability profile (111). The subsequent phase 3 trial of tirzepatide demonstrated more effective reductions in the glycated hemoglobin levels and body weight, along with greater overall improvements in the lipid profile compared to semaglutide in patients with T2DM, elevating incretin therapeutic agents to a new level (112). Tirzepatide became the first dual GLP/GLP-1 receptor agonist to gain FDA approval for treating T2DM in 2022 and obesity in 2023 [**Table 2**].

## 5 Pharmacological effects of GIP, GLP-1 and their mimetics

The pharmacological benefits of GIP, GLP-1, and their synthetic mimetics arise from their pleiotropic effects, encompassing at least five key functions: bolstering insulin secretion and survival of pancreatic β-cells and modulating glucagon release by the pancreatic α-cells in response to glycemic levels, acting on the satiety center in brain, slowing gastric emptying, regulating lipid and glucose metabolism through effects on adipose tissue and liver, and reducing systemic blood pressure.

### 5.1 GIP and GLP-1 effects on pancreas

#### 5.1.1 Effects on pancreatic β-cell insulin secretion and survival

Activation of either GIPR or GLP-1R on pancreatic β-cells initiates distinct yet overlapping downstream signaling cascades that ultimately amplify glucose-stimulated insulin secretion. Previous physiological studies have shown that both GIP (113–116) and GLP-1 (117) act in concert with glucose to enhance insulin gene transcription, mRNA stability, insulin biosynthesis, and insulin secretion. However, a significant decrease in insulinotropic activity was observed with GIP in diabetic patient with hyperglycemia, whereas relatively preserved activity was noted with GLP-1 in the same patients (58), suggesting divergent effects of GIP and GLP-1 on β-cells (**Figure 1** – Section B).

GIPRs are abundantly expressed on β-cells, and their activation induces a rise in cAMP and intracellular calcium levels, facilitating glucose-dependent insulin release from pancreatic β-cells (118). In a study involving 10 healthy male subjects, GIP infusion more than doubled the insulin secretion rate compared to the placebo group when the glucose was maintained at 216 mg/dL (35). Conversely, in another study with 10 healthy adults where glucose levels were maintained between 70 – 80 mg/dL, fat ingestion elicited a fivefold to sixfold rise in GIP levels without concomitant insulin secretion (119). These results suggest that mild to moderate hyperglycemia is required for the insulinotropic effect of GIP in nondiabetic subjects, acting as a mechanism to prevent reactive hypoglycemia following the consumption of fatty foods.

Similar to GIP, the stimulatory effects of GLP-1 on insulin expression and secretion were observed in the presence of high (450 mg/dL) but not normal (99 mg/dL) concentrations of glucose in the cultured cells, indicating the glucose-dependent insulinotropic effect of GLP-1 (117). The loss of GLP-1 effect on stimulating insulin secretion at basal plasma glucose concentrations limits the hypoglycemic risk even at high pharmacological concentrations of GLP-1 (28, 58). Marked insulinotropic effects have been observed in T2DM patients with a single injection of liraglutide (120) or acute infusion of exenatide (121). However, the long-term treatment with GLP-1RAs may reduce the insulin levels in both T2DM (122) and prediabetic patients (123), owing to the glycemic normalization and increased insulin sensitivity in these patients.

Pancreatic β-cell mass is significantly reduced by more than 50% in patients with T2DM, with lesser reductions already occurring in the prediabetes state (124). GIP and GLP-1 may exert long-term effects on β-cell survival and proliferation, potentially delaying the development of and even reversing T2DM. Studies conducted on various cellular and animal models have demonstrated that both GIP and GLP-1 exert marked anti-apoptotic effects in pancreas (125–127). GIP treatment has been shown to reduce glucolipotoxicity-induced cell death in C57 BL/6 and Bax^-/-^ pancreatic islets, but not GIPR^-/-^ pancreatic islets of the mouse models (114). A study by Wang et al. (128) in Wistar rats demonstrated that continuous subcutaneous infusion of GLP-1 reversed age-dependent decline in β-cell functions. In a study comparing the effects of GIPR and GLP-1R activation on β-cell survival in response to streptozotocin challenge in mice, Maida et al. (126) observed that GLP-1R signaling exerted more robust control of β-cell survival, relative to GIPR activation. Furthermore, exendin-4 has been found to stimulate β-cell mass expansion and pancreas regeneration in a partial pancreatectomy rat model of T2DM (129). However, the potential for delaying or preventing the progression of T2DM in patients treated with GIP and GLP-1 remains uncertain, as there is currently no definitive evidence from human studies demonstrating that enhanced incretin activity directly affects β-cell mass or alters the progressive loss of insulin secretory capacity in humans.

#### 5.1.2 Effects on pancreatic α-cell glucagon release

In addition to their insulinotropic effects on β-cells, both GIP and GLP-1 play a role in regulating glucagon secretion of pancreatic α-cells, with GIP increasing and GLP-1 suppressing glucagon secretion, both in a glucose-dependent manner. GIPRs have been localized to human pancreatic α-cells, and intravenous GIP infusion activates GIPR and intracellular signaling in pancreatic α-cells, augmenting glucagonotropic effects in hypoglycemic conditions in humans (130). During hyperglycemia, GIP potentiates glucose-induced insulin secretion without exhibiting a glucagonotropic effect (35). Based on collective human data, a glycemic threshold of 99 – 108 mg/dL may exist, below which GIP primarily exerts glucagonotropic actions (35). Through its dual insulinotropic and glucagonotropic effects on stabilizing glucose levels, GIP could provide a buffer against hyperglycemia when large amounts of glucose are consumed and mitigate reactive hypoglycemia, especially after meals with high fat content. The safeguard against reactive hypoglycemia is evolutionally crucial, as central nervous tissue relies on stable blood glucose levels (**Figure 1** – Section B).

On the contrary, GLP-1 suppresses glucagon secretion in both healthy individuals and those with T2DM under normoglycemia and hyperglycemia (58) but not during hypoglycemia (131), thereby reducing the potential for developing severe hyperglycemia or hypoglycemia. GLP-1’s inhibition of glucagon secretion appears to be indirectly triggered via paracrine effects in the islets. Evidence from experiments employing somatostatin receptor 2 (SSRT2) antagonists and SSRT2 knockout mice strongly suggests that GLP-1’s inhibitory actions on α-cells are indirect and partially mediated through somatostatin-dependent mechanisms (132). Somatostatin, secreted from pancreatic γ-cells, acts as an inhibitory paracrine to suppress glucagon release from pancreatic α-cells, and specific blockade of SSRT2 eliminates the inhibitory effect of GLP-1 on glucagon secretion. Insulin has also been suggested to play a significant role as a paracrine regulator of glucagon secretion (the intra-islet hypothesis), although the mechanism by which this inhibition occurs is not well elucidated (133). It has been demonstrated that the insulinotropic effect on β-cells and glucagonostatic effect on α-cells each contribute to about half of GLP-1’s blood glucose-lowering activity (134).

In an *in vitro* study, tirzepatide elicited a cAMP response in a human pancreatic β-cell line that was significantly higher than that observed for either GLP-1 or GIP alone, indicating a synergistic or additive activation of signaling in pancreatic β-cells by GIP and GLP-1 (63). A mechanism of action phase 1 study showed that the glycemic efficacy of tirzepatide in T2DM results from concurrent improvements in key components of diabetes pathophysiology, including β-cell function, insulin sensitivity, and glucagon secretion (135). These effects were substantial and help elucidate the remarkable glucose homeostasis abilities of tirzepatide observed in SURPASS-2 trial when compared with semaglutide (112).

### 5.2 GIP and GLP-1 effects on the central nervous system

The drive to forage and the regulation of satiety regarding nutrient intake are modulated by orexigenic and anorexigenic signaling pathways, respectively. These pathways are mainly located in arcuate nucleus of the hypothalamus (ARH). Neurons co-expressing orexigenic neuropeptide Y (NYP) and agouti-related peptide (AgRP) in the ARH activate melanocortin hormone- and orexin-expressing neurons in the lateral thalamus, leading to increase food intake. Conversely, satiety is primarily controlled by anorexigenic pro-opiomelanocortin (POMC) and cocaine- and amphetamine-regulated transcript (CART) neurons in the ARH that project to paraventricular nucleus neurons, resulting in the release α-melanocyte-stimulating hormone and a decrease of food intake. Modern foods, with their high caloric intensity, pose a challenge. The amount of oral intake needed to achieve gastric and intestinal fullness and signal satiety to the brain through the gut-brain axis can overwhelm the evolutionarily determined metabolic capacity of peripheral organs such as liver, pancreas, fat tissue, and muscles. This imbalance may lead to disruptions in glucose and lipid homeostasis, contributing to a range of metabolic diseases, including obesity. Incretins and their synthetic mimetics offer benefits by influencing the appetite and satiety centers in the brain, potentially resetting the body’s fat mass and body weight setpoints that are associated with the interplay between orexigenic and anorexigenic signaling pathways.

A study using *in situ* hybridization and polymerase chain reaction techniques did not detect GIP expression in the brain of adult male Sprague-Dawley rats (41). However, GIP immunoreactivity was identified in the adult rat brain using a specific monoclonal antibody against GIP (136), suggesting that while GIP is not expressed in the brain, peripheral GIP can reach the deep brain. GIPR was found in both neuronal and nonneuronal cells within the satiety and feeding centers of the hypothalamus in mice (137). Chronic central (intracerebroventricular) or peripheral (subcutaneous) infusion of GIP analogue in mice increased cFos neuronal activity in hypothalamic feeding center, resulting in reduced food intake and body weight. These effects were blunted in CNS-specific GIPR knockout mice, suggesting a key role of CNS GIPR in the control of energy metabolism (138) (**Figure 1** – Section C).

Unlike GIP, the fully processed GLP-1 is produced by the neurons within the nucleus of the solitary tract (NST) in the brainstem of rats (139). The expression of pre-proglucagon gene and GLP-1 has been detected in the human brainstem and hypothalamus (140). GLP-1 is a well-characterized neurotransmitter involved in signaling satiety in the brain (71). Neurons producing GLP-1 project to targets in the hypothalamus, where GLP-1Rs are abundantly expressed. GLP-1Rs are also found in circumventricular organs (CVO), the hind brain, and the thalamus. The presence of GLP-1R in the phylogenetically oldest parts of the brain but not in the cortex emphasizes that GLP-1 is involved in the regulation of vital functions like feeding and satiety (141).

Brain GLP-1Rs, rather than peripheral GLP-1Rs, appear crucial for the weight-reducing effects of GLP-1RAs. Studies in mice with CNS neuron-specific GLP-1R knockout showed that liraglutide failed to induce weight loss but still improved glucose levels, suggesting that brain GLP-1Rs are necessary for the weight-lowering effect of GLP-1RAs but not essential for their glucose-lowering effects (142). The ability of peripherally circulating GLP-1 and GLP-1RAs to breach the blood-brain barrier remains uncertain; they may potentially do so via the relatively permeable CVO or through endocytosis and transcytosis of vascular endothelial cells to reach the deep brain (143–145). Salameh et al. (146) observed significant blood-to-brain influx for non-acylated GLP-1 mimetics like exendin-4 and lixisenatide, whereas acylated GLP-1 analogues such as liraglutide and semaglutide showed minimal blood-brain barrier penetration in mice. Interestingly, Gabery et al. (145) demonstrated that peripherally administered liraglutide and semaglutide accessed ARH directly without crossing the blood-brain barrier. However, semaglutide exerted broader effects, extending laterally and more deeply into the posterior ARH compared to liraglutide, potentially explaining its greater effectiveness in weight reduction (147). Emerging evidence suggests that GLP-1RAs may reduce food intake and body weight through diffuse brain effects rather than targeting a specific localized population of GLP-1Rs (145).

Notably, the amount of incretin mimetics entering the brain parenchyma does not correlate with their efficacy in weight reduction (146). There are other mechanisms by which circulating incretin mimetics can influence deep brain function without crossing the blood-brain barrier. Peripherally circulating GLP-1 (143) and some GLP-1RAs, such as liraglutide and semaglutide (145), can readily access the CVOs, including the subfornical organ and the area postrema, which express GLP-1R and have close neuroanatomical connections with hypothalamus involved in forage and satiety control. While both CVO and the hypothalamus are important, the hypothalamus is likely more central to the direct regulation of appetite and satiety through GLP-1 signaling. The role of CVO is crucial for sensing peripheral GLP-1 levels and relaying this information to the hypothalamus and other deep brain regions. Most pharmacokinetic studies assessing the brain uptake of incretin mimetics have been conducted in animals, and translating these findings to human subjects and clinical outcomes remains uncertain.

In the brain, the two incretins are not redundant and may complement each other in regulating orexigenic and anorexigenic pathways (148). Studies in mice suggest that GIP may potentiate the GLP-1-induced reduction of food intake and body weight by enhancing POMC expression and neuronal activation within distinct neuron populations in the hypothalamus (149). Importantly, preclinical studies have demonstrated that co-administration of GIP and GLP-1 intracerebroventricularly results in an additive enhancement in weight-lowering efficacy (138). The potential mechanisms through which GLP-1 and GIP/GLP-1 receptor agonists recalibrate and normalize the fat mass and body weight setpoint in patients with obesity may stem from their primary effects on incretin homeostasis. These effects include neurotrophic, neurogenic, neuroprotective, anti-inflammatory, and insulin sensitizing actions, a field currently under intensive investigation and not yet fully understood.

### 5.3 GIP and GLP-1 effects on gastric emptying

The secretion of endogenous GIP does not correlate with gastric half-emptying times (150). Neither acute nor chronic exposure to GIP or GIPRAs affects gastric emptying in healthy individuals or those with T2DM (151). Conversely, exogenous infusion of GLP-1 exhibits potent inhibitory effects of gastric emptying, indicating an enterogastrone mechanism. In a study with eight poorly controlled T2DM patients who received a liquid meal via a nasogastric tube and exogenous infusion of GLP-1 to achieve plasma levels of about 70 pM, gastric volume remained constant over 2 hours, and plasma glucose returned to normal fasting values within 3 – 4 hours (152). Counteraction of GLP-1 effect on gastric emptying by co-infusion with prokinetic drug erythromycin during a liquid test meal diminished GLP-1’s ability to decrease postprandial hyperglycemia in patients with T2DM (153). The dose-dependent effect of GLP-1 on decelerating gastric emptying plays a substantial role in improving postprandial glycemic concentrations to the extent that postprandial insulin secretion may no longer be stimulated or could even be reduced (154, 155) (**Figure 1** – Section D).

Pharmacologically, only short-acting GLP-1RAs demonstrate a marked reduction in gastric emptying (156), while chronic activation of GLP-1R results in a waning effect on gastric emptying due to tachyphylaxis. This tachyphylaxis occurs with continuous infusion of GLP-1 (154, 157), long acting GLP-1RAs (156), or tirzepatide (151). Tachyphylaxis is contingent upon a full 24-hour exposure, as evidenced by a study in which rats treated with liraglutide (with half-life of 11 – 15 hours) twice daily for 14 days had markedly diminished gastric emptying while those treated with exenatide (with half-life of 2 – 3 hours) twice daily over the same period still displayed a profound reduction in gastric emptying, although both compounds had similar effects on body weight reduction (158). This phenomenon is further illustrated by results from a randomized, open-label mechanistic trial in patients with T2DM, which demonstrated that lixisenatide, with a half-life of 2 – 4 hours, had a significantly greater effect than liraglutide in delaying in gastric emptying and curtailing postprandial glucose excursions following a standardized solid breakfast (156).

GLP-1 has been shown to attenuate meal-induced gastric antral propagated contractions while increasing pyloric tone (159, 160). The experimental data from rats suggest that the inhibitory effect of GLP-1 on gastric motor function is mediated by the afferent vagal nerve, through interaction with GLP-1Rs located in vagal afferent fibers that transmit sensory signals to the brain (161). The brain regions (e.g., NST) responsible for mediating the inhibitory effect of GLP-1 on gastric emptying depend on intact vagal afferent input. In addition, the brain regions involved in mediating the effects of GLP-1 on gastric emptying and satiety differ, given that the threshold dose of GLP-1 required to inhibit gastric emptying is 3,000 to 10,000 times lower than the reported threshold doses necessary to suppress feeding behavior (161). GLP-1 may exert its effects locally via the vagus nerve before being degraded by DPP-4 in circulation. Studies in rats have identified GLP-1R expression in both the nodose ganglion (162) and vagal nerve terminals innervating the portal vein (163), and GLP-1 has been shown to increase impulse generation in both gastric and hepatic vagal afferent fibers (164, 165). Unlike GLP-1, GIP has not been found to possess a similar neural signaling mechanism for decelerating gastric emptying (54, 162).

The most common side effect of GLP-1RAs is nausea and vomiting, which are hypothesized to result from inhibition of gastric emptying. Long-acting agents seem to have a lower incidence of gastrointestinal adverse effects (166), likely due to tachyphylaxis in gastric emptying with sustained GLP-1R stimulation. Nonetheless, residual effects on gastric emptying could still produce significant gastrointestinal adverse effects in patients treated with long-acting GLP-RAs (167). The absence of GIP’s effect on gastric emptying underscores the physiological and pharmacological distinctions between GIP and GLP-1. Notably, the undesirable gastrointestinal effects associated with GLP-1RAs have not been observed with GIPRAs (168). Instead, GIP may counteract nausea and vomiting via direct modulation of the area postrema/NST emesis circuitry (169) and exert protective effects on unfavorable altered gut motility (44).

### 5.4 GIP and GLP-1 effects on lipid and glucose metabolism in adipose tissue and liver

Fat and liver are two organs that play key roles in lipid and glucose metabolism, working together in concert and complimentarily to maintain lipid and glucose homeostasis. Both GIP and GLP-1 exert direct or indirect effects on adipose and hepatic tissues to regulate lipid and glucose metabolism.

#### 5.4.1 Effects on adipose tissue

GIPRs are abundant in adipose tissue (170). In a study utilizing a cultured mouse embryo preadipocyte cell line, GIP was shown to enhance the synthesis and release of lipoprotein lipase (LPL) into the medium, thereby facilitating the uptake of triglycerides into the adipocytes (171). Additionally, Ebert et al. (172) demonstrated that intravenous infusion of porcine GIP abolished plasma hypertriglyceridemia in rats during the fat load, while rats pretreated with GIP antiserum exhibited a significantly greater triglyceride increment late in the fat load time course. GIP promotes clearance of circulating triglycerides and free fatty acids and enhances adipose tissue triglyceride absorption, serving as a crucial hormonal regulator of postprandial triglyceride response in humans (173–175). Moreover, GIP has been found to boost glucose uptake by improving insulin sensitivity in adipocytes (176, 177), while tirzepatide has been demonstrated to enhance insulin sensitivity and promote glucose disposal in white adipose tissue in Glp-1r-null mice (178), further supporting a direct effect of GIP on adipose tissues. The anabolic effects of GIP enhance both the postprandial glucose and lipid buffering capacity of white adipose tissue and prevent ectopic fat deposition in visceral organs (179, 180). The anabolic effects of GIP may play a physiologically permissive role for adipose tissue synthesis, and GIP antagonist antibodies have been developed as pharmacological agents for obesity treatment (68, 74) (**Figure 1** – Section E).

In contrast to GIPRs, GLP-1Rs are not expressed in adipose tissue (170). Existing data suggest that the effects of GLP-1 on adipose tissue are primarily indirect. GLP-1 may modulate lipid metabolism through increased sympathetic tone, leading to increased lipolysis, heightened triglyceride catabolism, and enhanced fatty acid oxidation (181). GLP-1Rs are densely expressed in the area postrema in rats (182). As the area postrema lacks the blood-brain barrier, it serves as a key site for peripheral GLP-1 to activate central autonomic regulatory sites. The targets of area postrema neuronal projections include the rostral ventrolateral medulla (RVLM), with the efferent projections from the RVLM innervating the sympathetic preganglionic neurons (183). These projections from the area postrema to the RVLM may constitute potential pathways activated by GLP-1 to elicit sympathetic responses. The sympathetic nerves release norepinephrine, which activates receptors on adipocytes, leading to the mobilization of stored triglycerides. Intriguingly, in patients with dumping syndrome, postprandial serum catecholamine rises correspond with increased serum GLP-1 levels (184). The increased sympathetic tone may explain a known GLP-1RA class effect of a modest but significant increase in heart rate, the clinical consequences of which are undetermined.

GIP promotes the proper storage of lipids in adipose tissue, whereas the robust fat oxidation mechanism of GLP-1 increases the utilization of these deposited lipids. The opposing effects of GIP and GLP-1 on lipogenesis and lipolysis, respectively, help maintain adipocytes in young and healthy state, fostering higher levels of adiponectin – a key adipokine known for its potential antidiabetic, anti-inflammatory, and antiatherogenic properties (185). Both GIP (186) and GLP-1 (187) enhance adiponectin expression and secretion in murine models. Elevated adiponectin concentrations have been noted in major clinical trials with GLP-1RAs (123, 188) and, more robustly, with tirzepatide (189). In a *post hoc* analysis of the SURPASS-1 trial involving patients with T2DM, the percentage increase in adiponectin levels from baseline ranged from 16% to 23% with tirzepatide compared to a decrease of 0.2% with placebo at week 40 (190).

#### 5.4.2 Effects on liver

Despite the lack of expression of GIPR (41, 191) and GLP-1R (192, 193) in hepatocytes, both GIP and GLP-1 have indirect effects on hepatic lipid and glucose metabolism (194). The secretion of adiponectin from adipose tissue enhanced by both incretins elicits a signal akin to a fasting state in hepatocytes through the cAMP/pAMPK pathway. This signaling pathway upregulates enzymes such as carnitine palmitoyl transferase 1, a key enzyme in β-oxidation, while concurrently reducing the expression of lipogenesis-related genes, including SREBP-1c and FAS (195). Adiponectin also decreases hepatic *de novo* glucose production and reduces free fatty acid levels in the liver (196), thereby mitigating hepatic steatosis, enhancing insulin sensitivity, and improving the circulating lipid profile (**Figure 1** – Section E).

Moreover, in addition to the direct action of GIP in adipose tissue to reduce serum triglyceride levels, GLP-1 may abolish the postprandial rise in triglyceride and free fatty acid concentrations by reducing intestinal absorption of dietary lipid, stemmed from the delayed gastric emptying (197, 198). Both GLP-1RAs and tirzepatide demonstrated efficacy in improving circulatory lipid profiles in the large clinical trials (199, 200). Intriguingly, a *post hoc* analysis of a phase 2 clinical trial revealed that dulaglutide treatment resulted in reductions in apoC-III and apoB levels without impacting serum LPL level, whereas treatment with tirzepatide showed a dose-dependent increase in serum LPL activity while concurrently reducing apoC-III and apoB levels (180).

Elevated levels of triglycerides and free fatty acids in circulation are major factors driving lipid accumulation in the liver, leading to metabolic dysfunction-associated steatohepatitis (MASH) and liver fibrosis. MASH contributes to worsening insulin resistance and is associated with an increased risk of cardiovascular disease. Clinical outcome studies have demonstrated the therapeutic potential of GLP-1RAs and tirzepatide in mitigating MASH. In small proof-of-concept phase 2 randomized controlled trials (RCTs) in patients with biopsy-confirmed MASH, liraglutide at 1.8 mg/day for 48 weeks (201), semaglutide at 0.4 mg/day for 72 weeks (202), and tirzepatide at 15 mg/week for 52 weeks (203) have been reported to achieve placebo-corrected percentages of participants meeting the criteria for resolution of MASH without worsening fibrosis by 30% (P = 0.019), 42% (P < 0.001), and 52% (P < 0.001), respectively. Additionally, the placebo-corrected percentages of participants with an improvement of at least one fibrosis stage without worsening MASH were 3% (P = 0.46), 10% (P = 0.48), and 21% (95% CI, 1 – 42), respectively. Treatment periods longer than 48 to 72 weeks may be needed to show substantial treatment effects on fibrosis with GLP-1 and dual GIP/GLP-1 receptor agonists. The effects of semaglutide on regression of fibrosis in patients with MASH are currently being investigated in the ongoing phase 3 ESSENCE trial (ClinicalTrials.gov number, NCT 04822181).

### 5.5 GLP-1 effects on systemic blood pressure

Intracerebroventricular GLP-1 injection stimulated the urinary excretion of water and sodium in a preclinical study of rats (72). GLP-1Rs have also been detected in kidney vasculature and tubular epithelial cells, potentially reducing the expression of fibrotic and inflammatory mediators in the kidney and decreasing sodium and fluid reabsorption in the proximal tubule (204). Liraglutide has been shown to enhance atrial natriuretic peptide secretion in mice (205), and this cardiac hormone reduces blood pressure mediated by natriuresis and vasodilatation. GLP-1RAs have been shown to increase natriuresis and diuresis in both healthy volunteers and individuals with obesity or diabetes. Exenatide has been demonstrated to diminish sodium intake in individuals with obesity without affecting salt craving (206). Gutzwiller et al. (207) reported that GLP-1 infusion evoked a dose-dependent increase in urinary sodium excretion in both healthy subjects and insulin-resistant men with obesity. Additionally, Lovshin et al. (208) reported that three weeks of liraglutide treatment significantly increased both 24-hour and nighttime urinary sodium excretion in hypertensive subjects with T2DM.

Consequently, chronic treatment with GLP-1RAs or tirzepatide improves both systolic and diastolic blood pressures, often occurring before significant weight loss is observed (209, 210). Data from a subset of 494 participants showed individuals receiving 10 mg/week of tirzepatide had lower placebo-adjusted systolic blood pressure and diastolic blood pressure by 10.6 mmHg and 2.9 mmHg, respectively, in ambulatory blood pressure monitoring at week 36 (211). Importantly, they do not reduce blood pressure in normotensive subjects, and hypotension has not been associated with GLP-1RAs and tirzepatide in clinical trials. Understanding how GLP-1R signaling coupled to blood pressure reduction is restricted or silenced in normotensive subjects is an important area for future investigation. On the other hand, the effects of GIP on blood pressure are not documented.

## 6 Clinical applications of GLP-1RAs and tirzepatide

Current treatment guidelines for T2DM have evolved from a narrow “glucose-centric” focus to addressing the underlying causes of disease and the associated detrimental health consequences, particularly adiposity and cardiovascular complications. Originally developed for the treatment of T2DM, GLP-1RAs and tirzepatide have shown promise in promoting weight loss and attenuating the risk of cardiovascular diseases.

### 6.1 Clinical applications in T2DM

In terms of glucose control effectiveness, most GLP-1 analogues outperform exendin-4 analogues [**Supplementary Table**]. Exendin-4-based agents, including exenatide IR (10 μg twice daily) (212, 213), exenatide ER (2 mg/week) (214), and lixisenatide (20 μg/day) (215, 216), all reduced glycated hemoglobin of less than 1% compared to placebo in the pivotal phase 3 RCTs conducted at their highest doses approved by the FDA. However, the placebo-adjusted reduction in glycated hemoglobin was 0.8% in the HARMONY 1 trial of albiglutide (30 mg/week) (217), 1.05% in the AWARD 1 trial of dulaglutide (1.5 mg/week) (218), 1.34% in the LEAD-1 SU trial of liraglutide (1.8 mg/day) (219), 1.5% in the SUSTAIN 1 trial of subcutaneous semaglutide (1.0 mg/week) (220), and 1.1% in the PIONEER 1 trial of oral semaglutide (14 mg daily) (221), respectively, when administered at doses of the upper limit approved by the FDA for the treatment of T2DM. Among GLP-1RAs, only liraglutide has been approved for use in adolescent population (age 10 or above) with T2DM [**Table 2**]. In the SURPASS-1 trial (222), the patients of T2DM treated with tirzepatide at 15 mg/week achieved a placebo-corrected glycated hemoglobin reduction by 2.11%, demonstrating superior efficacy compared to currently approved GLP-1RAs.

The reduction in glycated hemoglobin was primarily driven by lowering postprandial glucose with short-acting GLP-1RAs (e.g., exenatide-4 IR and lixisenatide), and a greater reduction in fasting blood glucose levels with a carryover effect on postprandial glucose following treatment with long-acting GLP-1RAs and tirzepatide (166, 223). In trials comparing the addition of injectable agents in individuals requiring further glucose lowering, glycemic efficacy of GLP-1RAs and tirzepatide was found to be similar or greater than that of basal insulin (224–226). GLP-1RAs and tirzepatide in these trials demonstrated a lower hazard of hypoglycemia, albeit with a higher incidence of gastrointestinal side effects. Consequently, these trials have led to the recommendation of GLP-1RAs and tirzepatide as the preferred agents for individuals in need of potency provided by an injectable therapy for glucose control (227).

### 6.2 Clinical applications in weight management

Weight loss was consistently observed in nearly all landmark clinical trials of GLP-1RAs and tirzepatide. Based on a series of comparison studies in patients with T2DM, the agents at their highest approved doses could be roughly ranked from least to most efficacy in terms of weight reduction as follows: albiglutide (50 mg/week) ≤ lixisenatide (20 μg/day) ≤ exenatide IR (10 μg twice a day) = exenatide ER (2 mg/week) ≤ dulaglutide (1.5 mg/week) < liraglutide (3.0 mg/day) < injectable semaglutide (2.4 mg/week) < tirzepatide (15 mg/week) [**Supplementary Table**]. The range of placebo-corrected weight loss varied from 0.83 kg (95%CI, -1.06 to -0.60) with albiglutide (50 mg/week) at 16 months in the Harmony Outcomes trial (228) to 11.6 kg with tirzepatide (15 mg/week) in the 72-week SURMOUNT-2 trial (229) in patients with T2DM. However, it’s important to approach indirect comparisons between trials with caution due to variations in study designs, durations, populations, and analysis methods. These studies ranged in duration from 24 weeks to 72 weeks, and it is unclear whether the relative efficacy of these agents would differ when used for longer durations.

Studies have indicated that albiglutide (228, 230) and dulaglutide (231) were not as effective as liraglutide and semaglutide in generating weight loss in head-to-head comparisons. These findings are consonant with the larger molecular structures of both albiglutide and dulaglutide, which limit their ability to reach the deep brain and thus have minimal effects on the brain’s satiety center. Only three injectable incretin analogues – liraglutide (dose up to 3.0 mg/day, marketed as Saxenda), injectable semaglutide (dose up to 2.4 mg/week, marketed as Wegovy), and tirzepatide (dose up to 15 mg/week, marketed as Zepbound) – are approved by the FDA as adjuncts to lifestyle modifications, including diet and exercise interventions, for weight loss. Additionally, both liraglutide and semaglutide are approved for indefinite use in weight management in adolescent aged 12 years and older (232) [**Table 2**].

A series of trials assessing the efficacy of GLP-1RAs and tirzepatide in reducing weight in patients, both with or without a diagnosis of T2DM, included the SCALE trials for liraglutide, the STEP trials for semaglutide, and the SURMOUNT trials for tirzepatide. Subcutaneous administration of liraglutide (3.0 mg/day) for 56 weeks, semaglutide (2.4 mg/week) for 68 weeks, or tirzepatide (15 mg/week) for 72 weeks resulted in placebo-corrected weight loss of 4.0% in the SCALE Diabetes study (122), 6.2% in the STEP 2 study (233), and 9.6% in the SURMOUNT-2 (229) study, respectively, in patients with T2DM. In contrast, they achieved more substantial weight loss of 6.0% in the SCALE Obesity and Prediabetes study (123), 12.4% in the STEP 1 study (234), and 17.8% in the SURMOUNT-1 study (200), respectively, in patients without diabetes. All three agents demonstrated reduced efficacy in individuals with T2DM, resulting in 30% to 50% less body weight reduction compared to those without diabetes. Moreover, in studies involving patients with T2DM, the magnitude of body-weight loss with GLP-1RAs diminished as baseline hyperglycemia worsened (235, 236). Based on these findings, it is hypothesized that the heightened insulin resistance might render patients more resistant to the weight-reducing effects of GLP-1RAs and tirzepatide.

Even modest reductions in body weight, ranging from 3 to 7%, can improve glycemic control and mitigate cardiometabolic risk factors, while more substantial weight loss (>10%) may lead to diabetes remission (237). Indeed, it has been suggested that agents capable of achieving an average weight reduction of about 15% in individuals with obesity represent a new generation of anti-obesity medications, as this level of weight loss is sufficient to combat or prevent a broader range of obesity-related complications (238). In the SURMOUNT-2 trial involving 938 adults with a BMI of 27 kg/m^2^ or higher and T2DM, up to 65%, 48%, and 31% of participants on tirzepatide 15 mg/week experienced body weight reductions of ≥ 10%, ≥ 15%, and ≥ 20%, respectively, by week 72, with 49% of those treated with tirzepatide achieving normoglycemia (glycated hemoglobin < 5.7%) compared to only 3% treated with placebo (229).

Participants treated with liraglutide (239), semaglutide (240), or tirzepatide (200) in the clinical trials had a percent reduction in fat mass approximately three times greater than the reduction in lean mass, leading to an overall improvement in body composition. The ratio of fat-mass loss to lean-mass loss was similar to that reported in lifestyle-based intervention in the Look AHEAD trial (241) and surgical treatment for obesity (242). However, loss of lean muscle mass could be potentially detrimental to patients, especially those who are older or have chronic illnesses. Ongoing research into nutritional and exercise interventions, as well as novel pharmacological agents – such as myostatin/activin pathway inhibitors (243) or agents targeting the apelin pathway (244) – holds promise for preserving lean mass and improving overall health in patients undergoing treatment with GLP-1 and dual GIP/GLP-1 receptor agonists.

In patients treated with GLP-1RAs and tirzepatide, gastrointestinal side effects such as nausea and vomiting are common but do not significantly contribute to weight loss. Although patients receiving exenatide IR and experiencing longer durations of nausea and vomiting tended to lose more weight, those in the trial group who did not experience the gastrointestinal symptoms (70%) generally lost weight as well (166, 224). A *post hoc* subgroup analysis assessing changes in BMI standard-deviation scores between participants with one or more gastrointestinal adverse events and those with none revealed that the effect of liraglutide in reducing BMI standard-deviation scores was independent of gastrointestinal adverse events (P = 0.82) (245). A previous mediation analysis of semaglutide in adults with overweight or obesity suggested that less than one percentage point of the body weight reduction achieved with subcutaneous semaglutide was attributable to gastrointestinal adverse events (246). The analysis of the SURPASS trials of tirzepatide revealed no association between weight reduction and gastrointestinal adverse events (229).

### 6.3 Clinical applications in cardiovascular disease prevention

Recent American College of Cardiology/American Heart Association guidelines advocate for the use of GLP-1RAs in patients with T2DM at high risk of atherosclerotic cardiovascular disease (ASCVD), irrespective of well-controlled glucose levels, based on the clinical trials mandated by FDA-issued industry guidelines in December 2008 to assess the cardiovascular effects of new diabetic therapies (247). As of 2022, GLP-1RAs have been recommended as first-line treatments, along with SGLT-2 inhibitors, for patients with T2DM and cardiovascular disease in American Diabetes Association’s Standards of Medical Care in Diabetes (248, 249). However, not all tested GLP-1RAs have consistently demonstrated reductions in cardiovascular events, and the impact on specific cardiovascular outcomes varied among the effective drugs.

Eight major RCTs of GLP-1RAs have investigated the cardiovascular outcomes, including the ELIXA trial of lixisenatide (250), the EXSCEL trial of exenatide ER (251), the LEARDER trial of liraglutide (252), the SUSTAIN-6 trial of subcutaneous semaglutide (253), the HARMONY trial of albiglutide (228), the REWIND trial of dulaglutide (254), the PIONEER trial of oral semaglutide (255), and the SELECT trial of subcutaneous semaglutide (199). They were all large-scale trials involving 3,000 to 18,000 patients who were either at high risk of or having established ASCVD, with trial durations ranging from 15 to 60 months. The first seven trials involved patients with T2DM and baseline glycated hemoglobin levels ranging from 7.3 to 8.7, whereas the most recent SELECT trial focused on nondiabetic patients with overweight or obesity and preexisting cardiovascular disease. All these trials were designed to evaluate the primary outcome of 3-MACE defined as cardiovascular death, nonfatal myocardial infarct, and nonfatal stroke, except for the ELIXA trial of exenatide ER, which included hospitalization for unstable angina as an additional component of the 4-MACE composite outcomes.

All eight reported studies have demonstrated the noninferiority of GLP-1RAs compared to placebo regarding MACE. Additionally, the superiority of GLP-1RAs compared to placebo in the primary endpoint of 3-MACE were found with liraglutide in the LEADER trial (HR 0.87; 95% CI 0.78 – 0.97; P = 0.01), injectable semaglutide in the SUSTAIN trial (HR 0.74; 95%CI 0.58 – 0.95; P = 0.02) and in SELECT trial (HR 0.80; 95% CI 0.72 – 0.90; P < 0.001), albiglutide in the HARMONY trial (HR 0.78; 95% CI 0.68 – 0.90; P = 0.0006), and dulaglutide in REWIND trial (HR 0.88; 95% CI 0.79 – 0.99; P = 0.026). The REWIND trial suggested that dulaglutide might be effective for both primary and secondary cardiovascular prevention in individuals with T2DM (254). Notably, significant reductions of death from any causes were shown in both the LEADER trial with liraglutide (HR 0.85; 95% CI 0.74 – 0.97; P = 0.02) (252) and the SELECT trial with semaglutide (HR 0.81; 95% CI 0.71 – 0.93) (199). A significant benefit in reducing death from cardiovascular causes was only shown in the LEADER trial with liraglutide (HR 0.78; 95% CI 0.66 – 0.93; P = 0.007). The recent FLOW trial with semaglutide in patients with chronic kidney disease and T2DM, with a composite of major kidney disease events as the primary outcome, showed significant reductions in both cardiovascular mortality (HR 0.71; 95% CI 0.56 – 0.89) and all-cause mortality (HR 0.80; 95% CI 0.67 – 0.95) in the confirmatory secondary outcomes, in addition to the reduced risk of the primary outcome events (HR 0.76; 95% CI 0.66 – 0.88; P = 0.0003) (256). Following the withdrawal of albiglutide from the market in 2018 by the manufacturer for financial reasons (257), liraglutide, injectable semaglutide, and dulaglutide should currently be the preferred choices within this class for reducing cardiovascular events until further data are available (248).

In the SUSTAIN-6 trial, injectable semaglutide demonstrated a notable effect in reducing the incidence of nonfatal stroke (hazard ratio [HR] 0.61; 95% CI 0.38 – 0.99; P = 0.04), a main contributor to the reduction of 3-MACE composite endpoint (253). Similarly, the REWIND trial showed a stronger impact of dulaglutide on nonfatal stroke (HR 0.76; 95% CI 0.61 – 0.98; P = 0.017) compared to nonfatal myocardial infarction (HR 0.96; 95% CI 0.79 – 1.16; P = 0.65) and cardiovascular death (0.91; 95% CI 0.78 – 1.067; P = 0.21) (254). On the other hand, the reports from the LEADER trial of liraglutide showed a less significant hazard ratio for nonfatal stroke (HR 0.89; 95% CI, 0.72 – 1.11; P = 0.30) (252), and the HARMONY Outcomes trial of albiglutide indicated a similar trend in reducing total stroke (HR 0.86; 95% CI 0.66 – 1.14; P = 0.30) (228). Whether the superior effects of semaglutide and dulaglutide on stroke reduction compared to other GLP-1RAs arise from inherent differences in drug properties or disparities in trial design remains uncertain. Confirmatory large-scale trials are essential to ascertain the potential efficacy of GLP-1RAs in specifically improving the stroke outcomes.

In addition to GLP-1RAs, pioglitazone and SGLT-2 inhibitors are two other classes of diabetic medications that have shown promise in reducing the risk of cardiovascular disease, albeit through different mechanisms. Pioglitazone represents another potentially important therapy for preventing stroke events (258). Unlike treatment with GLP-1RAs, which generally leads to body weight and fat mass reduction, pioglitazone dose-dependently increases body weight and fat mass. Paradoxically, this increase has been associated with improved all-cause mortality as well as cardiovascular mortality, according to a *post hoc* analysis of the PROactive trial (259). However, the benefit in reducing stroke events was not seen in the outcome studies of SGLT-2 inhibitors (260). Unlike GLP-1RAs, particularly dulaglutide (254), SGLT-2 inhibitors have not demonstrated cardiovascular benefits in primary prevention among individuals with multiple cardiovascular risk factors (261).

The SUPRPASS-4 trial (262), involving 2002 participants with high-risk cardiovascular profiles followed for up to 104 weeks, showed no difference in 4-MACE events (cardiovascular death, myocardial infarction, stroke, and hospitalization for unstable angina) with tirzepatide compared to insulin glargine (HR 0.74; 95% CI, 0.51 – 1.08), the latter having been demonstrated to have neutral effect on cardiovascular outcomes (263). A meta-analysis of seven SURPASS clinical trials in participants with T2DM showed the hazard ratios comparing tirzepatide versus controls of 0.80 (95% CI, 0.57 – 1.11) for 4-MACE, 0.90 (95% CI, 0.50 – 1.61) for cardiovascular death, and 0.80 (95% CI, 0.51 – 1.25) for all-cause mortality (264). The definitive impact of tirzepatide on cardiovascular disease will be addressed in the ongoing SURPASS-COVT study (ClinicalTrials.gov number, NCT04255433), which compares tirzepatide with dulaglutide in patients with T2DM and ASCVD. The study will provide a more comprehensive assessment of the cardiovascular outcomes of this dual GIP/GLP-1 receptor agonist.

The mechanisms underlying the favorable cardiovascular effects of GLP-1RAs remain incompletely understood. These benefits are independent of baseline glycemia and duration of diabetes and only minimally related to their glucose-lowering efficacy (254). Although the pathophysiological processes underlying the cardiovascular disease may improve with weight loss, weight loss alone is insufficient to fully account for the observed cardiovascular improvements. Clinical trials of GLP-1RAs have primarily focused on patients with obesity or overweight, yet the effectiveness in the cardiovascular protection appears independent of baseline BMI range (265). Furthermore, the cardiovascular benefits emerged early in treatment, preceding significant weight loss, suggesting that the cardiovascular effects may be mediated, at least partially, by other salutary processes in atherosclerotic protection. Treatment with GLP-1RAs leads to rapid reduction in blood pressure, which, according to a meta-analysis (266), could substantially lower the risk of cardiovascular disease and mortality. Additional factors such as increased release of adiponectin from adipose tissue, amelioration of insulin resistance in liver and peripheral tissues, and improved circulatory lipid profile all contribute to the reduced risk of ASCVD (267). Recent findings indicate that the anti-inflammatory effects of GLP-1RAs, mediated through GLP-1R in CNS neurons (268) and intrahepatic lymphocytes (269), play an important role in their antiatherogenic actions. The FLOW trial also highlights the renal protective effects of GLP-1RAs, suggesting a close link between renal and cardiovascular benefits, which warrants further mechanistic studies (256).

## 7 Conclusion and challenges

GLP-1RAs have been used to treat T2DM since 2005 and gained approval for weight management in 2014. In studies on cardiovascular diseases, GLP-1RAs have shown potential benefits. Notably, in high-quality large RCTs, tirzepatide demonstrated greater effectiveness than GLP-1R mono agonists in reducing serum glucose, fat mass, and body weight. Ongoing clinical trials will provide clarity on the presumed cardiovascular benefits of tirzepatide. However, the global supply of these medications remains limited, often resulting in a first-come, first-served distribution. To address this, there have been proposals to prioritize individuals at higher risk of premature death, aligning with ethical principles of equitable care (270). Nevertheless, it is crucial to consider the potential adverse effects of weight fluctuation after discontinuing these medications, particularly the increased risk of cardiovascular and all-cause mortality in patients with obesity or overweight.

For all their promise, GLP-1 and dual GIP/GLP-1 receptor agonists have raised more questions, particularly regarding their long-term safety implications. Clinical trials have provided limited insight into the long-term effects, as the longest trials spanned less than six years in adults and about one year in adolescents. Given the natural fluctuations in hormone levels and the intricate rhythms of human physiology throughout the day and over a person’s lifetime, there is concern about the uncertain health ramifications of sustained elevation of incretin activity over decades of treatment. Like virtually all medications, incretin mimetics come with side-effects, leading some patients to abandon treatment. Common adverse effects include nausea and vomiting, as well as increased hazards of gastroparesis and small bowel obstruction. The next generation of regimens may involve combining GLP-1 or dual GIP/GLP-1 receptor agonists with other nutrient-based hormone receptor agonists or antagonists that offer equal or greater effectiveness with fewer gastrointestinal adverse effects.

## Author contributions

QL: Conceptualization, Data curation, Formal analysis, Writing – original draft, Writing – review & editing.
