# Supplementary material for: Mechanisms of action and therapeutic applications of GLP-1 and dual GIP/GLP-1 receptor agonists
Source: Front Endocrinol (Lausanne). 2024 Jul 24;15:1431292. doi: 10.3389/fendo.2024.1431292 (PMC11304055; doi:10.3389/fendo.2024.1431292)
Supplement: Supplementary file 1 [file DataSheet_1.pdf]

Supplemental Table: Phase 3 Clinical Trials Comparing the Effectiveness of Various GLP-1RAs and Tirzepatide in Reducing Glycated Hemoglobin and Body Weight

Clinical trial (Duration of trial)	Treatment	Change of glycated hemoglobin		Weight loss	
		Efficacy	Estimated treatment difference	Efficacy	Estimated treatment difference
GetGoal-X (24 weeks) (1)	Lixisenatide (20 µg daily)	-0.79% (SE ± 0.05%)	0.17% (95% CI, 0.033% – 0.297%)	-2.96 kg (SE ± 0.23 kg)	1.02 kg (95%CI, 0.456 kg to 1.581 kg)
	Exenatide IR (10 µg twice a day)	-0.96% (SE ± 0.05%)		-3.98 kg (SE ± 0.23 kg)	
DURATION-1 (30 weeks) (2)	Exenatide ER (2 mg weekly)	-1.9% (SE ± 0.1%)	-0.4% (95% CI, -0.54 to -0.12%)	-3.7 kg (SE ± 0.5)	-0.1 kg (95% CI, -1.3 kg to 1.1 kg)
	Exenatide (10 µg twice a day)	-1.5% (SE ± 0.1%)		-3.6 kg (SE ± 0.5)	
LEAD-6 (26 weeks) (3)	Liraglutide (1.8 mg daily)	-1.12% (SE ± 0.08)	-0.33 (95%CI -0.47 to -0.18)	-3.24 kg (SE ± 0.33)	-0.38 kg (95% CI -0.99 to 0.23)
	Exenatide (10 µg twice a day)	-0.79% (SE ± 0.08)		-2.87 kg (SE ± 0.33)	
AWARD-1 (26 weeks) (4)	Dulaglutide (1.5 mg weekly)	-1.51% (SE ± 0.06%)	Dulaglutide 1.5 mg vs. Exenatide: -0.52% (95% CI -0.66 to -0.39)<br><br>Dulaglutide 0.75 mg vs. Exenatide: -0.31% (95%CI -0.44 to -0.18)	-1.30 kg (SE ± 0.29)	Dulaglutide 1.5 mg vs. Exenatide: -0.24 kg [P < 0.001]<br><br>Dulaglutide 0.75 mg vs. Exenatide: 1.27 kg (P < 0.001))
	Dulaglutide (0.75 mg weekly)	-1.30% (SE ± 0.06%)		0.20 kg (-1.07 kg (SE ± 0.29)	
	Exenatide (10 µg twice a day)	-0.99% (SE ± 0.06%)		-1.07 kg (SE ± 0.29)	
DURATION-6 (26 weeks) (5)	Exenatide ER (2 mg weekly)	-1.28% (SE ± 0.05%)	0.21% (95% CI, 0.08 to 0.33)	-2.68 kg (SE ± 0.18)	0.90 kg (95%CI 0.39 to 1.40)
	Liraglutide (1.8 mg daily)	-1.48% (SE ± 0.05%)		-3.57 kg (SE ± 0.29)	
HARMONY 7 (32 weeks) (6)	Albiglutide (50 mg weekly)	-0.78% (95%CI -0.87 to -0.69)	0.21% (95% CI 0.08 to 0.34)	-0.64 kg (95%CI -1.00 to -0.28)	1.55 kg (95%CI 1.05 to 2.06)
	Liraglutide (1.8 mg daily)	-0.99% (95%CI -1.08 to -0.90)		-2.19 kg (95%CI -2.55 to -1.83)	
AWARD-6 (26 weeks) (7)	Dulaglutide (1.5 mg weekly)	-1.42% (SE ± 0.05)	-0.06% (95% CI -0.19 to 0.07)	-2.90 kg (SE ± 0.22)	0.71 kg (95% CI 0.17 to 1.26)
	Liraglutide (1.8 mg daily)	-1.36% (SE ± 0.05)		-3.61 kg (SE ± 0.22)	
SUSTAIN 7 (40 weeks) (8)	Semaglutide (0.5 mg weekly)	-1.5% (SE ± 0.06)	Semaglutide 0.5 mg vs. dulaglutide 0.75	-4.6 kg (SE ± 0.28)	Semaglutide 0.5 mg vs. dulaglutide 0.75 mg: -

	Semaglutide (1.0 mg weekly)	-1.8% (SE ± 0.06)	mg: -0.4% (95% CI -0.55 to -0.25)<br><br>Semaglutide 1.0 mg vs. dulaglutide 1.0 mg: -0.41% (95%CI -0.57 to -0.25)	-6.5 kg (SE ± 0.28)	2.26 kg (95% CI -3.02 to -1.51)<br><br>Semaglutide 1.0 mg vs. dulaglutide 1.0 mg: -3.55 kg (95%CI -4.32 to -2.78)
	Dulaglutide (0.75 mg weekly)	-1.1% (SE ± 0.05)		-2.3 kg (SE ± 0.27)	
	Dulaglutide (1.5 mg weekly)	-1.4% (SE ± 0.06)		-3.0 kg (SE ± 0.27)	
SUSTAIN 10 (30 weeks) (9)	Semaglutide (1.0 mg weekly)	-1.7%	-0.69% (95%CI -0.82 to -0.56)	-5.8 kg	-3.83 kg (95%CI -4.57 to -3.09)
	Liraglutide (1.2 mg daily)	-1.0%		-1.9 kg	
SUSTAIN 3 (56 weeks) (10)	Semaglutide (1.0 mg weekly)	-1.5% (SE ± 0.06)	-0.62% (95%CI -0.80 to -0.44)	-5.6 kg (SE ± 0.29)	-3.78 kg (95%CI -4.58 to -2.98)
	Exenatide ER (2.0 mg weekly)	-0.9% (SE ± 0.06)		-1.9 kg (SE ± 0.29)	
PIONEER 4 (26 weeks) (11)	Oral semaglutide (14 mg daily)	-1.2% (SE ± 0.1)	Oral semaglutide vs. liraglutide: -0.1% (95%CI -0.3 to -0.0)	-4.4 kg (SE ± 0.2)	Oral semaglutide vs. liraglutide: -1.2 (95%CI -1.9 to -0.6)
	Liraglutide (1.8 mg daily)	-1.1% (SE ± 0.1)		-3.1 kg (SE ± 0.2)	
	Placebo	-0.2% (SE ± 0.1)		-0.5 kg (SE ± 0.3)	
PIONEER 10 (52 weeks) (12)	Oral semaglutide (3 mg daily)	-0.9% (SE ± 0.1)	Oral semaglutide 14 mg vs. dulaglutide: -0.3% (95%CI -0.6 to -0.1)	0.0 kg (SE ± 0.3)	Oral semaglutide 14 mg vs. dulaglutide: -2.6 kg (95%CI -2.6 to -1.6)
	Oral semaglutide (7 mg daily)	-1.4% (SE ± 0.1)		-0.9 kg (SE ± 0.3)	
	Oral semaglutide (14 mg daily)	-1.7% (SE ± 0.1)		-1.6 kg (SE ± 0.3)	
	Dulaglutide (0.75 mg weekly)	-1.4% (SE ± 0.1)		1.0 kg (SE ± 0.4)	
SURPASS-2 (40 weeks) (13)	Tirzepatide (5 mg weekly)	-2.01%	Tirzepatide 5 mg vs. Semaglutide 1 mg -0.15% (95% CI -0.28 to -0.03)	-7.6 kg	Tirzepatide 5 mg vs. Semaglutide 1.0 mg -1.9 kg (95% CI -2.8 to -1.0)
	Tirzepatide (10 mg weekly)	-2.24%		-9.3 kg	
	Tirzepatide (15 mg weekly)	-2.30%		-11.2 kg	
	Semaglutide (1.0 mg weekly)	-1.86%		-5.7 kg	
AWARD-11 (52 weeks) (14)	Dulaglutide (1.5 mg/week)			-3.5 kg	
	Dulaglutide (3.0 mg/week)			-4.3 kg	

	Dulaglutide (4.5 mg/week)			-5.0 kg	
STEP 2 (68 weeks) (15)	Semaglutide (2.4 mg/week)	-1.6%		-9.6% (-9.5 kg)	2.4 mg vs. placebo: -6.2% (-7.3 to -5.2)<br><br>2.4 mg vs. 1.0 mg: -2.7% (-3.7 to -1.6)
	Semaglutide (1.0 mg/week)	-1.5%		-7.0% (-6.9 kg)	
	Placebo	-0.4%		-3.4% (-3.4 kg)	
STEP 8 (68 weeks) (16)[1]	Semaglutide (2.4 mg/week)	-0.2% (-0.3 to -0.2)		-15.8% (95%CI -17.6 to -13.9) -15.3 kg	Semaglutide vs. liraglutide: -9.4% (-12.0 to -6.8)
	Liraglutide (3.0 mg/day)	-0.1% (-0.1 to 0.0)		-6.4% (95%CI -8.2 to -4.6) -6.8 kg	
	Pooled placebo			-1.9%	

1, study of 338 overweight or obese adults without diabetes.

References

1. Rosenstock J, Raccah D, Korányi L, Maffei L, Boka G, Miossec P, et al. Efficacy and safety of lixisenatide once daily versus exenatide twice daily in type 2 diabetes inadequately controlled on metformin: a 24-week, randomized, open-label, active-controlled study (GetGoal-X). Diabetes Care. 2013;36(10):2945-51.
2. Drucker DJ, Buse JB, Taylor K, Kendall DM, Trautmann M, Zhuang D, et al. Exenatide once weekly versus twice daily for the treatment of type 2 diabetes: a randomised, open-label, non-inferiority study. Lancet. 2008;372(9645):1240-50.
3. Buse JB, Rosenstock J, Sesti G, Schmidt WE, Montanya E, Brett JH, et al. Liraglutide once a day versus exenatide twice a day for type 2 diabetes: a 26-week randomised, parallel-group, multinational, open-label trial (LEAD-6). Lancet. 2009;374(9683):39-47.
4. Wysham C, Blevins T, Arakaki R, Colon G, Garcia P, Atisso C, et al. Efficacy and safety of dulaglutide added onto pioglitazone and metformin versus exenatide in type 2 diabetes in a randomized controlled trial (AWARD-1). Diabetes Care. 2014;37(8):2159-67.
5. Buse JB, Nauck M, Forst T, Sheu WH, Shenouda SK, Heilmann CR, et al. Exenatide once weekly versus liraglutide once daily in patients with type 2 diabetes (DURATION-6): a randomised, open-label study. Lancet. 2013;381(9861):117-24.
6. Pratley RE, Nauck MA, Barnett AH, Feinglos MN, Ovalle F, Harman-Boehm I, et al. Once-weekly albiglutide versus once-daily liraglutide in patients with type 2 diabetes inadequately controlled on oral drugs (HARMONY 7): a randomised, open-label, multicentre, non-inferiority phase 3 study. Lancet Diabetes Endocrinol. 2014;2(4):289-97.

7. Dungan KM, Povedano ST, Forst T, González JG, Atisso C, Sealls W, et al. Once-weekly dulaglutide versus once-daily liraglutide in metformin-treated patients with type 2 diabetes (AWARD-6): a randomised, open-label, phase 3, non-inferiority trial. Lancet. 2014;384(9951):1349-57.

8. Pratley RE, Aroda VR, Lingvay I, Lüdemann J, Andreassen C, Navarria A, et al. Semaglutide versus dulaglutide once weekly in patients with type 2 diabetes (SUSTAIN 7): a randomised, open-label, phase 3b trial. Lancet Diabetes Endocrinol. 2018;6(4):275-86.

9. Capehorn MS, Catarig AM, Furberg JK, Janez A, Price HC, Tadayon S, et al. Efficacy and safety of once-weekly semaglutide 1.0mg vs once-daily liraglutide 1.2mg as add-on to 1-3 oral antidiabetic drugs in subjects with type 2 diabetes (SUSTAIN 10). Diabetes Metab. 2020;46(2):100-9.

10. Ahmann AJ, Capehorn M, Charpentier G, Dotta F, Henkel E, Lingvay I, et al. Efficacy and Safety of Once-Weekly Semaglutide Versus Exenatide ER in Subjects With Type 2 Diabetes (SUSTAIN 3): A 56-Week, Open-Label, Randomized Clinical Trial. Diabetes Care. 2018;41(2):258-66.

11. Pratley R, Amod A, Hoff ST, Kadowaki T, Lingvay I, Nauck M, et al. Oral semaglutide versus subcutaneous liraglutide and placebo in type 2 diabetes (PIONEER 4): a randomised, double-blind, phase 3a trial. Lancet. 2019;394(10192):39-50.

12. Yabe D, Nakamura J, Kaneto H, Deenadayalan S, Navarria A, Gislum M, et al. Safety and efficacy of oral semaglutide versus dulaglutide in Japanese patients with type 2 diabetes (PIONEER 10): an open-label, randomised, active-controlled, phase 3a trial. Lancet Diabetes Endocrinol. 2020;8(5):392-406.

13. Frías JP, Davies MJ, Rosenstock J, Pérez Manghi FC, Fernández Landó L, Bergman BK, et al. Tirzepatide versus Semaglutide Once Weekly in Patients with Type 2 Diabetes. N Engl J Med. 2021;385(6):503-15.

14. Bonora E, Frias JP, Tinahones FJ, Van J, Malik RE, Yu Z, et al. Effect of dulaglutide 3.0 and 4.5 mg on weight in patients with type 2 diabetes: Exploratory analyses of AWARD-11. Diabetes Obes Metab. 2021;23(10):2242-50.

15. Davies M, Færch L, Jeppesen OK, Pakseresht A, Pedersen SD, Perreault L, et al. Semaglutide 2·4 mg once a week in adults with overweight or obesity, and type 2 diabetes (STEP 2): a randomised, double-blind, double-dummy, placebo-controlled, phase 3 trial. Lancet. 2021;397(10278):971-84.

16. Rubino DM, Greenway FL, Khalid U, O'Neil PM, Rosenstock J, Sørrig R, et al. Effect of Weekly Subcutaneous Semaglutide vs Daily Liraglutide on Body Weight in Adults With Overweight or Obesity Without Diabetes: The STEP 8 Randomized Clinical Trial. JAMA. 2022;327(2):138-50.
